# Probing the effect of NEK7 and cofactor interactions on dynamics of NLRP3 monomer using molecular simulation

**DOI:** 10.1002/pro.4420

**Published:** 2022-09-21

**Authors:** Sherihan El‐Sayed, Sally Freeman, Richard A. Bryce

**Affiliations:** ^1^ Division of Pharmacy and Optometry, School of Health Sciences, Manchester Academic Health Sciences Centre University of Manchester Manchester UK; ^2^ Department of Medicinal Chemistry, Faculty of Pharmacy Zagazig University Zagazig Egypt

**Keywords:** ADP, ATP, molecular simulation, NEK7 interactions, NLRP3 inflammasome, pocket accessibility

## Abstract

The NLRP3 inflammasome is a cytoplasmic complex that regulates the activation of inflammatory cytokines and, given its implication in a range of diseases, is an important therapeutic target. The cofactor ATP and the centrosomal kinase NEK7 are important for NLRP3 activation. Here we have constructed and simulated computational models of full‐length monomeric NLRP3 to shed light on the importance of NEK7 and cofactor interactions for its conformation and dynamics in aqueous solution. We find that molecular dynamics simulation reproduces well the features of the recently published cryo‐EM structure of the ADP‐bound NLRP3–NEK7 complex; on the removal of NEK7, the NLRP3 molecule adopts a more compact closed form during simulations. Replacement of ADP by ATP promotes a rearrangement of hydrogen‐bonding interactions, domain interfaces, and a degree of opening of the NLRP3 conformation. We also examine the dynamics of an acidic loop of the LRR domain of NLRP3, which samples in a region observed in the NEK7‐bound cryo‐EM structure but not in an oligomeric form of inactive NLRP3. During the molecular dynamics simulations of NLRP3, we find some plasticity in its topology that suggests access routes for ATP to the cofactor pocket not immediately evident from the existing NEK7‐bound cryo‐EM structure. These computed dynamical trajectories of NLRP3 provide insight into coordinates of deformation that may be key for cofactor binding and inflammasome activation.

## 1 INTRODUCTION

**FIGURE 1:**
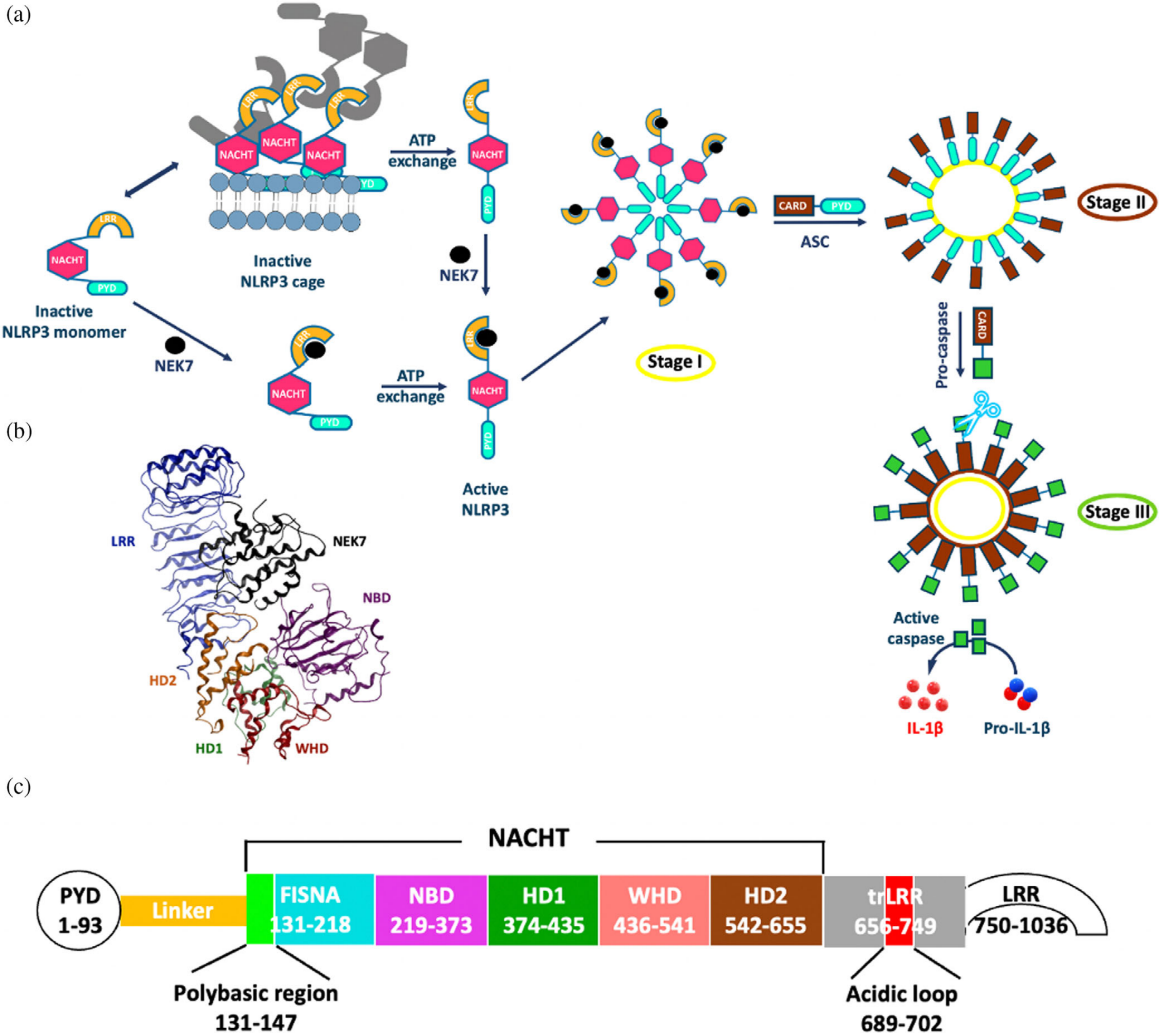
(a) Diagrammatic representation of NLRP3 inflammasome activation: stage I, sensor oligomerization; stage II, NLRP3^PYD^–ASC^PYD^ interaction and formation of ASC filaments; stage III, ASC^CARD^–procaspase^CARD^ interaction and activation of caspase. (b) Cryo‐EM structure of NEK7/NLRP3/ADP (PDB code 6NPY).^9^ (c) Domains in NLRP3 sequence

The inflammasome is a large multiprotein complex that assembles in response to pathogen‐associated molecular patterns (PAMPs) or damage‐associated molecular patterns (DAMPs). The resulting structure then is able to recruit adaptor protein ASC (apoptosis‐associated speck‐like protein) to form a speck, then activating caspase‐1, with subsequent release of interleukins and cell death (Figure 1a). Considerable effort has focused on elucidating the mechanism of inflammation given its linkage, when activated inappropriately, to a range of pathologies including cancers,^1^, ^2^ metabolic disorders,^3^ atherosclerosis,^4^, ^5^ and Alzheimer's disease.^6^

The best‐characterized inflammasome to date is that formed by the protein NLRP3, belonging to the NOD‐like receptor (NLR) family. NLRP3 consists of three domains: a N‐terminal pyrin (PYD) domain, a central NACHT domain, and a C‐terminal leucine‐rich repeat (LRR) domain (Figure 1b,c). The NACHT domain is composed of subdomains (Figure 1c), which includes the nucleotide‐binding domain (NBD); within the NBD, the Walker A site (Gln225–Ile234), Walker B site (Arg296–Ile315), sensor 1 motif (Leu346–Arg351), and sensor 2 motif (Pro365–Glu369) form the pocket in which cofactor ATP is bound.^7^ This cofactor is required for activation of NLRP3 before ASC polymerization by pyrin–pyrin interactions (Figure 1a). In its active state, NLRP3 is thought to comprise a disk‐like oligomer of NLRP3, stabilized by a single ring of NACHT–NACHT interactions, analogous to the activated form of NAIP‐NLRC4.^8^

One mechanism of NLRP3 activation has found the centrosomal Ser/Thr kinase NEK7 to play a key role,^9^ binding with nanomolar affinity to the LRR domain in interphase and promoting NLRP3–ASC‐caspase 1 assembly. The kinase can be in its active, P‐loop phosphorylated, state, or inactive form, for NLRP3 to be activated. Recently solved structures of NLRP3 from cryo‐EM crystallography have provided invaluable insights into inflammasome mechanism: for example, a cryo‐EM structure of monomeric human NLRP3, in complex with the interacting C‐lobe of NEK7, was solved by Sharif et al.^9^ This pyrin‐deleted structure exhibited the NLRP3 in an inactive conformation, complexed to its cofactor ADP as well as NEK7. The kinase interacted with the curved LRR domain and with the NBD and HD2 subdomains of the NACHT domain (Figure 1b).

Subsequently, a cryo‐EM structure of murine NLRP3 by Andreeva et al.^10^ revealed a multimeric structure for the inactive form of murine NLRP3. This double‐ring conformation comprised six to eight NLRP3 dimers associated via their LRR domains and precluded binding to NEK7. Some density was resolved for pyrin domains clustered within the center of this ring, but insufficient to permit structure assignment. Interestingly, a bioluminescence resonance energy transfer (BRET) study^11^ revealed a gradual structural opening of the inactive conformation of NLRP3 upon potassium efflux. Indeed, the fish‐specific NACHT‐associated domain (FISNA) region of NLRP3 (residues 131–218, Figure 1c) appears to sense low potassium and allows NLRP3 to adopt an active conformation. A polybasic sequence (residues 131–147), which overlaps with the FISNA, enables membrane interaction of NLRP3. It seems that cytosolic NLRP3 is monomeric or dimeric and membrane association via the polybasic region leads to oligomer formation. NLRP3 can activate in the absence of LRR, which does indicate that the cage is not an absolute requirement.^12^

A subsequent cryo‐EM structure of human NLRP3 in its inactive ADP‐bound form, complexed with the inhibitor MCC950, also known as CRID3, was consistent with this double‐ring structure.^13^ In this case, a pentamer of dimers was formed; the MCC950 compound occupied the Walker A site at the convergence of four subdomains of NLRP3. An acidic loop appeared to bridge LRR–LRR interface, binding into a basic concave region of LRR. This cryo‐EM structure complements a crystal structure of the NACHT domain of ADP‐bound NLRP3 in complex with an MCC950 analog (NP3‐146),^14^ confirming the role of the inhibitor as a form of “intramolecular glue”^14^ between subdomains. Very recently, two cryo‐EM structures of human and mouse NLRP3 in complex with MCC950^15^ were released in their inactive hexameric and dodecameric forms, respectively. In all oligomer structures, the same LRR‐binding interface pattern of “back to back” and “face to face” binding is observed.

These recent cryo‐EM and crystallographic structures, of monomeric and oligomeric NLRP3, provide valuable insights into inflammasome organization, yielding geometries of a range of inactive states at various levels of resolution. Molecular dynamics (MD) simulations can provide a complementary approach that gives dynamical insights into protein structure and interactions, revealing conformations and motions that may be of functional significance. Indeed, MD has been applied to study various aspects of NLRP3 previously, including NACHT domain rotation as a function of MCC950^16^, ^17^ and cofactor^18^, ^19^ and flexibility of the isolated NLRP3 pyrin domain,^20^ although these simulations were of limited duration.

In this work, we employ MD simulations to probe the effect of NEK7 and cofactor on NLRP3 monomer structure and dynamics, and consider the associated potential implications for inflammasome function. Specifically, based on its cryo‐EM structure, we construct, simulate, and validate a full‐length model of NLRP3 with NEK7 bound. From these calculations, we assess the flexibility of the complex and the NEK7‐NLRP3 interactions that underpin the nanomolar binding affinity of the complex. To characterize the degree to which NEK7 stabilizes a more open, active structure of NLRP3, we then remove NEK7 and evaluate changes in the global conformation of NLRP3. We employ MD and accelerated MD simulations^35^ to probe global changes in conformation, where either ADP or ATP is bound. We assess the impact of the change of cofactor in terms of local cofactor‐NLRP3 interactions as well as domain and global conformation. In doing so, we seek to identify structures and motions relevant to (in)activation of the NLRP3 molecule. The path by which cofactor exchange occurs through NLRP3 is not clear based on the existing experimentally determined 3D structures, so we also seek to identify potential routes for this process from the dynamical trajectories generated. In addition, we consider an acidic loop of the LRR domain of NLRP3: this loop has been found in one of the cryo‐EM structures of inactive NRLP3 to interact with a basic region of the LRR domain, although the functional significance of this interaction is unknown. Here, we examine the conformational behavior of this acidic loop for the NLRP3 systems simulated.

## 2 COMPUTATIONAL METHODS

### 2.1 NLRP3 model preparation

The full‐length NLRP3 model was constructed based on the 3.8 Å resolution cryo‐EM structure of ADP‐bound NLRP3/NEK7 by Sharif et al.^9^ (PDB code 6NPY).^9^ Homology modeling using SWISS‐MODEL server (https://swissmodel.expasy.org/) was used to account for the unresolved loops in the NACHT domain. We follow Sharif et al.^9^ in considering the C‐lobe of NEK7, which forms the interface with NLRP3; modeling has indicated that the N‐lobe projects away from the structure and was not included in the construct used for their cryo‐EM structure^36^ or in this work. The quality of the NLRP3^NACHT‐LRR^ model was evaluated and compared to the cryo‐EM structure, prior to loop addition, using Ramachandran plots (Figure S1), ProSA (protein structure analysis)^21^ (Figure S2), and 3D structure verification^22^ (Figure S3). These gave good metrics: for example, the *Z*‐score of the NLRP3^NACHT‐LRR^ model from ProSA^21^ is −7.5, which is more favorable than the value of −6.6 for the cryo‐EM^NACHT‐LRR^ structure (see Supporting Information for more information). The structure of the missing pyrin domain was adopted from the X‐ray structure of the isolated domain (PDB code 3QF2),^23^ which also contained part of the connecting linker. The remaining initial conformation of the linker, which incorporates some of the polybasic region, was modeled in an extended form and refined using a 100 ns implicit water simulation to allow efficient exploration of conformations. This was then attached to form the full‐length NLRP3/NEK7 monomer model for subsequent MD simulations. The initial models for simulation of ADP‐bound and ATP‐bound NLRP3 models were taken from an equilibrated timepoint of the NLRP3/NEK7 simulation (more details below).

### 2.2 Molecular dynamics simulations

Simulations were prepared and conducted using the AMBER 19 package.^24^ The simulation system for NLRP3/NEK7 was generated by embedding the complex in a cubic water box of 98,353 TIP3P^25^ water molecules. For ADP‐bound NLRP3 and ATP‐bound NLRP3, it became evident from the greater flexibility of NEK7‐free NLRP3 during MD that a larger box was prudent for these systems; thus, rather generous boxes of 284,590 and 285,134 water molecules were used for the ADP‐bound NLRP3 and ATP‐bound NLRP3 simulations, respectively. The solvated systems were neutralized by the addition of sodium ions. Sodium chloride was added at a physiological concentration (150 mM) to the neutralized systems.^26^ Force field parameters were taken from the *ff14SB* force field^27^ for the protein in combination with *gaff2* ^28^ and the AMBER database for ADP and ATP cofactors (http://amber.manchester.ac.uk/).^29^ The generated topology files were modified by *parmed* ^30^ to repartition the mass of heavy atoms into the bonded hydrogen atoms; this enabled the use of HMR (hydrogen mass repartitioning),^31^ with a time step of 4 fs. A nonbond cutoff of 9.0 Å was used, along with the particle mesh Ewald (PME)^32^ method for long‐range electrostatic interactions.

Before simulation, unfavorable interactions in the NLRP3/NEK7 model were relaxed by energy minimization. The minimized system was then heated gradually from 0 to 300 K using the Langevin thermostat,^33^ and then equilibrated at 300 K and 1 atm with a Berendsen barostat;^34^ in doing so, the entire protein complex was restrained for 50 ns and then the modeled loops were released from restraints during a further 50 ns. Then, the remaining protein restraints were released gradually over another 50 ns followed by 300 ns equilibration without restraints. For both ADP‐bound and ATP‐bound NLRP3, the protein and cofactor were restrained for 10 ns and then the restraints were released over 10 ns followed by 50 ns without restraints. Production MD for the three systems ran for 1 μs at 300 K. For the ADP‐bound NLRP3 simulation in the absence of NEK7, a conformation of the NLRP3/NEK7 trajectory at 700 ns was taken as the initial structure. NEK7 was removed from the system, and NLRP3 resolvated and reequilibrated at 300 K for 70 ns, before a 1 μs production simulation. For the ATP‐bound model, the same protocol was followed, with the additional modification of ADP to include the γ‐phosphate group. The cofactor and its immediate protein environment were optimized before simulation.

### 2.3 Accelerated molecular dynamics

Accelerated MD was used to extend conformational sampling of the ADP‐bound NLRP3 and ATP‐bound NLRP3 models by lowering the energy barriers in the simulation system.^35^ Here, when the potential *V*(***r***) of the model system falls below a threshold boost energy *E*, the simulation is performed using the modified potential V*(***r***) = *V*(***r***) + Δ*V*(***r***), where Δ*V*(***r***) is a boost potential function, given by Δ*V*(***r***) = (*E* − *V*(***r***))^2^/(α + (*E* – *V*(***r***))). Here, α is the parameter that determines the strength of the system acceleration. Dual boosting, of the total and dihedral potentials, was used to accelerate the ADP‐bound and ATP‐bound NLRP3 simulations. The values of *E* and α were calculated from the averaged total potential energy and dihedral energy obtained at the end of 1 μs of unbiased MD simulation (Table S1). These boost potentials were then applied to each system during a canonical AMD simulation of 600 ns at 300 K in explicit solvent starting from the final configuration of the corresponding 1 μs unbiased MD simulation.

### 2.4 Simulation analysis

The cpptraj^36^ program was used to analyze MD trajectories. Visualization used VMD 1.9.3,^37^ MOE 2020.09,^38^ and Chimera 1.13.1 programs.^39^ Principal component analysis (PCA) was applied to study the global motion in NLRP3 during both MD and AMD simulations. PCA was performed for protein Cα atoms using the pyPCAzip package.^40^ PCA was performed using trajectories of the last 500 ns of MD simulation as well as the last 300 ns of AMD. PCA of a trajectory of 50 ns MD of the restrained initial NLRP3/NEK7 model was used as a reference.

## 3 RESULTS

### 3.1 Construction and simulation of NLRP3/NEK7 model

A model of full‐length NLRP3 was first constructed, based on the cryo‐EM structure of NEK7 associated pyrin‐deleted NLRP3 monomer;^9^ missing loop regions were assigned by homology modeling (Section 2). The structure of the absent pyrin domain was adopted from the X‐ray structure of the isolated pyrin domain,^23^ which also contained part of the linker that connects to the NACHT domain (residues Trp94–Glu110). The remaining residues of the linker, which overlaps with the polybasic region (residues Asp111 to Lys134), were separately modeled: from an initially extended conformation, the linker was refined using a 100 ns implicit solvent simulation.^41^, ^42^ The final simulated structure was then attached to form the initial NLRP3/NEK7 monomer model, prior to a further equilibration (Section 2).

**FIGURE 2:**
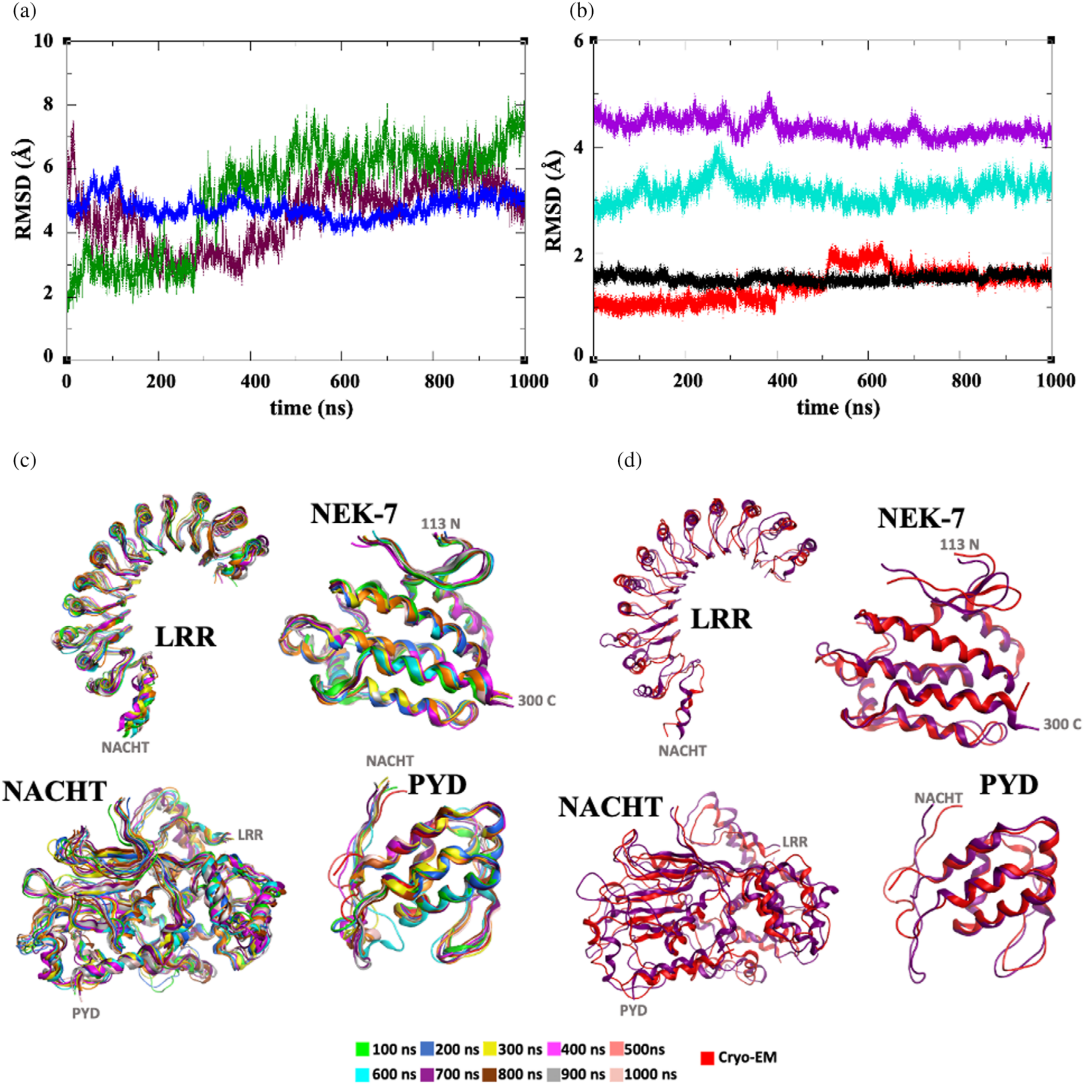
(a) Time series of RMSD of non‐pyrin backbone atoms in ADP‐bound NLRP3/NEK7 complex (blue), ADP‐bound NLRP3 (maroon), and ATP‐bound NLRP3 (green) over microsecond MD trajectory. (b) Time series of backbone RMSD of PYD (red), NACHT (purple), LRR (cyan), and NEK7 (black) regions of ADP‐bound NLRP3/NEK7 complex over microsecond MD trajectory. (c) Superposition of snapshots of domains of ADP‐bound NLRP3/NEK7 complex over microsecond MD trajectory. (d) Superposition of domains of the NLRP3/NEK7 complex at 700 ns from MD and from the cryo‐EM structure^9^

MD simulation of this NLRP3/NEK7 model was then performed for one microsecond in an explicit solvent at 300 K. After ~400 ns, the backbone root‐mean‐square deviation (RMSD) of NLRP3^NACHT‐LRR^ converges to a value of 4.8 Å from the cryo‐EM structure (blue, Figure 2a and Table S2). The RMSD of the individual domains however is rather lower, with values of 1.4, 4.4, and 3.2 Å for the pyrin, NACHT, and LRR domains of NLRP3, respectively; and an average RMSD value of 1.5 Å for NEK7 (Figure 2b and Table S2). Indeed, snapshots from the MD trajectory of the individual domains further illustrate the rather stable tertiary structures of the individual components over the 1 μs MD simulation (Figure 2c) and their very good agreement with the cryo‐EM conformation (Figures 2d and S4).

**FIGURE 3:**
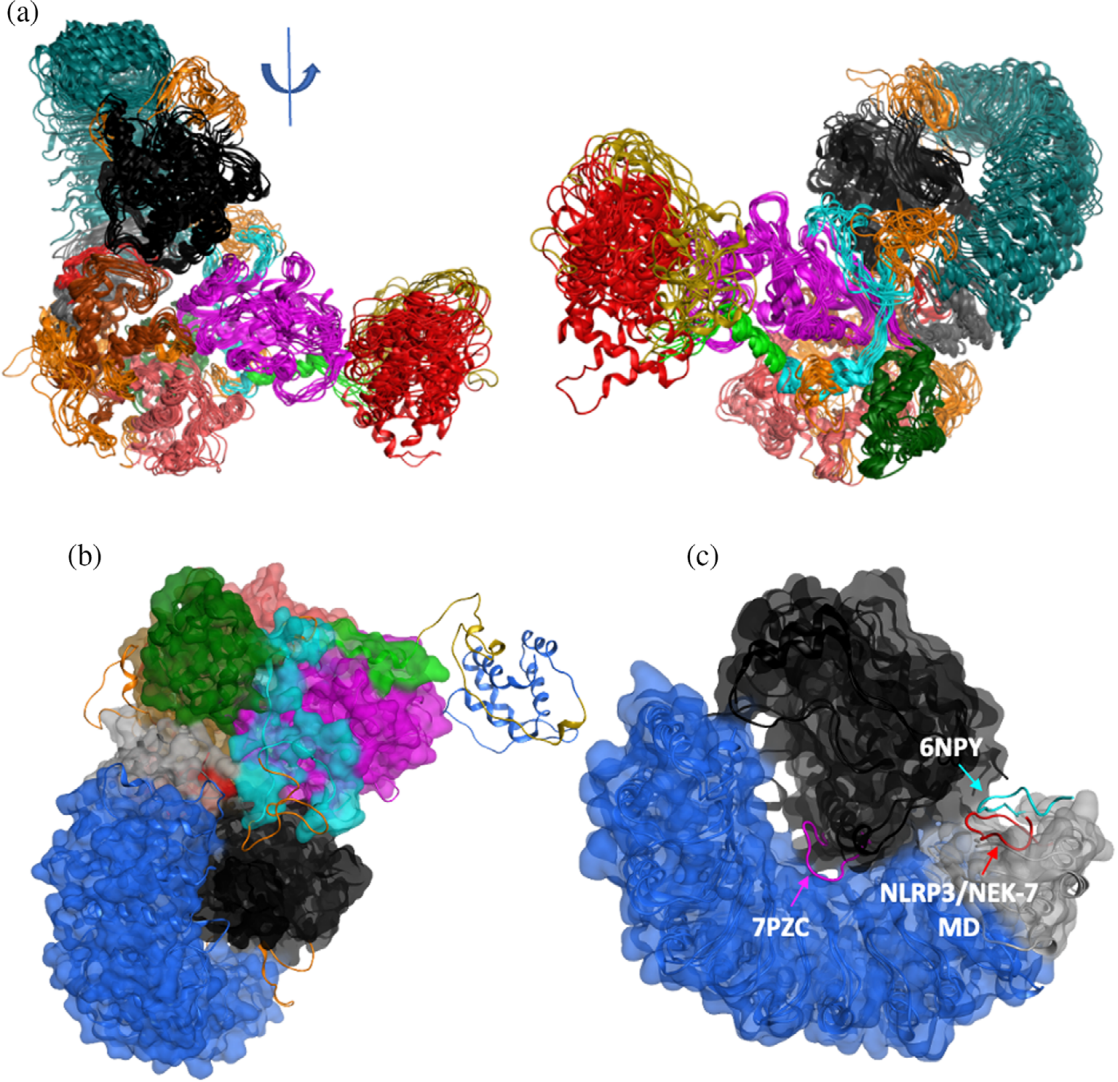
(a) Two views of superimposed equispaced snapshots of NLRP3/NEK7 complex from microsecond MD simulation, indicating PYD (red), NACHT (using a color scheme of Figure 1c), LRR (turquoise), NEK7 (black), and modeled unresolved loops (orange). (b) Final structure of NLRP3/NEK7 complex from microsecond MD simulation (ribbon), overlaid on cryo‐EM structure (space‐filling); no structure was assigned in the latter published structure for PYD. (c) Comparison of acidic loop geometry from NLRP3/NEK7 simulation (red) with that in cryo‐EM monomer^9^ (cyan) and in oligomer (magenta) structures.^13^ Cryo‐EM monomer complex in surface representation

**FIGURE 4:**
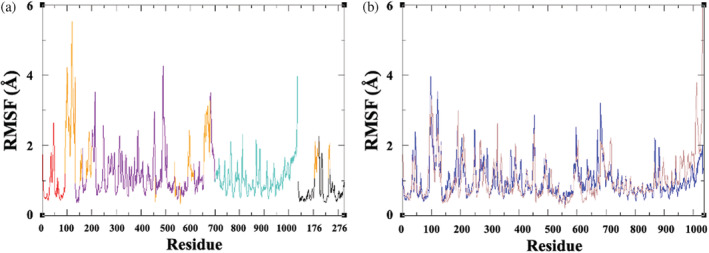
(a) Backbone RMSF as a function of residue for NLRP3/NEK7 complex over microsecond MD simulation, denoting PYD (red), NACHT (purple), LRR (cyan), NEK7 (black), and modeled unresolved loops (orange). (b) Backbone RMSF of NLRP3 with (blue) and without (brown) NEK7 bound, over the last 500 ns of MD trajectory

To evaluate the mobility of protein structural elements, we may also consider the root mean square fluctuation (RMSF) in atomic position, averaged over residue: as expected, several of the regions of high RMSF are associated with amino acids in modeled loops that were unresolved in the cryo‐EM structure (orange, Figures 3a,b and 4a). This includes part of the polybasic region (residues Lys131–Lys134), as well as flexible loops of the FISNA region (Asp153–Val162 and Ser179–Pro202). Indeed, there is a range of conformations explored by the pyrin domain relative to the rest of the NLRP3 structure (Figure 3a). There are also relatively high RMSF values for the C‐terminal region of the LRR domain (Figure 3a), although the overall LRR structure appears to form a more regular β‐sheet ladder than that observed in the cryo‐EM structure (Figure S5). Finally, we note for NEK7 a region of high RMSF which corresponds to the P‐loop, again an unresolved feature in the cryo‐EM structure. Nevertheless, the structure fits well within the envelope of the cryo‐EM structure (Figure 3b).

Despite these regions of high mobility, NEK7 and NLRP3 remain firmly bound to one another, consistent with an observed nanomolar dissocation constant.^9^ The NEK7‐binding NLRP3 interface is formed by residues in the NBD, HD2, and LRR domains; the observed cryo‐EM polar and hydrophobic interactions with these domains are well maintained over the course of the MD simulation (Tables S3–S5). The polybasic region is solvent exposed (green, Figure 3a). We also note that the acidic loop of the trLRR subdomain (residues Lys689 to Asp702), although somewhat mobile, remains in the vicinity of its cryo‐EM conformation (Figure 3c). Indeed, the presence of NEK7 would appear to preclude the significant displacement of the loop required to occupy the region observed in the cryo‐EM structure of NLRP3 bound to MCC950 by Hochheiser et al. (7PZC in Figure 3c);^13^ there the acidic loop interacts electrostatically with a basic concave region of LRR, comprising residues Ser749, Arg774, Arg779, Lys831, Arg859, Lys888, His916, Arg920, and Lys973. We note that the orientation of the acidic loop in the latter structure may arise from an unexpected rearrangement, with residue mismatches facilitating the interaction of this acidic loop with LRR. This mismatch appears to be absent in other cryo‐EM structures of NLRP3 in complex with MCC950.^10^, ^15^

In regard to the ADP cofactor of NLRP3, we note that the nucleotide maintains its hydrogen‐bonding interactions with the NBD, HD1, and WHD subdomains of the NACHT domain over the course of the MD simulation (Figure S6a,b and Table S6). However, as has been observed^11^ for the parent inactive cryo‐EM structure of ADP‐bound NLRP3/NEK7,^9^ no clear access channel through which ADP could exchange with ATP is apparent during the microsecond MD simulation.

### 3.2 Simulation of ADP‐bound NLRP3 model without NEK7

**FIGURE 5:**
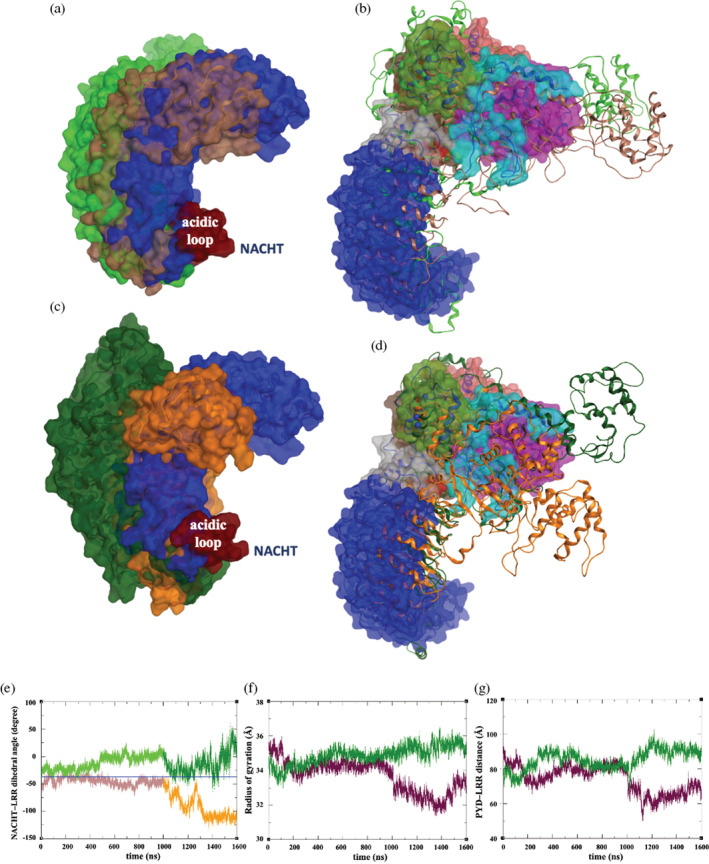
(a) Comparison of the LRR domain orientation (when NACHT domain is superimposed) of final structures ADP‐bound NLRP3/NEK7 (blue), ADP‐bound NLRP3 (brown), and ATP‐bound NLRP3 (green) from microsecond MD simulation. (b) Comparison of the cryo‐EM structure of ADP‐bound NLRP3/NEK7 (space‐filling) with final structures from microsecond MD simulation of ADP‐bound NLRP3 (brown ribbon) and ATP‐bound NLRP3 (green ribbon). (c) Comparison of the LRR domain orientation (when NACHT domain is superimposed) of final structures from MD simulation of ADP‐bound NLRP3/NEK7 (blue) and AMD simulations of ADP‐bound NLRP3 (orange) and ATP‐bound NLRP3 (dark green). (d) Comparison of the cryo‐EM structure of ADP‐bound NLRP3/NEK7 (space‐filling) with final structures from AMD simulation of ADP‐bound NLRP3 (orange ribbon) and ATP‐bound NLRP3 (dark green ribbon). (e) Time series of NACHT–LRR dihedral angle (see text for definition) over a combined 1,600 ns MD/AMD simulation of ADP‐bound NLRP3 (brown/orange) and ATP‐bound NLRP3 (green/dark green). Blue line indicates the average dihedral value from MD of NLRP3/NEK7. (f) Time series of the radius of gyration of NLRP3 over a combined 1,600 ns MD/AMD simulation of ADP‐bound (maroon) and ATP‐bound NLRP3 (dark green). (g) Time series of PYD–LRR distance (between C_α_ of residues Leu57 and Asn950) over a combined 1,600 ns MD/AMD simulation of ADP‐ (maroon) and ATP‐bound NLRP3 (dark green)

We now consider the structural and dynamical effects on NLRP3 when NEK7 is absent. A representative conformation was taken from the trajectory of the NLRP3/NEK7 complex and reequilibrated in the absence of NEK7 (Section 2). This was followed by a microsecond production MD simulation. The backbone RMSD of NLRP3^NACHT‐LRR^ converges after ~500 ns to a value of 4.5 Å (maroon, Figure 2a). As found in the NLRP3/NEK7 simulation, the internal geometries of the pyrin and NACHT domains remain similar to the cryo‐EM structure (Figure S7 and Table S7). However, the RMSD of the LRR domain progressively increases, with an average value of 3.3 Å (Figure S7). This is similarly reflected in the RMSF values, which for the last 36 residues of the LRR region are significantly higher than for the NEK7 complex (Figure 4b). Inspection of the trajectory indicates that, in the absence of NEK7, a rotation of the LRR domain occurs with respect to the NACHT domain (brown vs. blue, Figure 5a,b).

We characterize this rotation, a coordinate of possible significance in the (in)activation of NLRP3, using a NACHT‐LRR dihedral angle defined by the C_α_ atoms of residues Val223 (NBD), Ala394 (HD1), Leu932 (LRR), and Glu1019 (LRR). The average value of this angle over the final 500 ns of NLRP3/NEK7 simulation was −33°, which is very close to the value of −37° in the cryo‐EM structure. For the NEK7‐free NLRP3/ADP simulation, however, we observe a shift to an average value of −48° over the last 600 ns MD (Figure 5e). Associated with this NACHT‐LRR rotation is a slight decrease in the radius of gyration (Figure 5f). To further probe the extent of this rotation, we applied accelerated MD to the final NEK7‐free NLRP3 simulation structure for an additional 600 ns at 300 K. We observe further rotation of the structure (orange, Figure 5c,d), to an average dihedral value of −110° over the last 200 ns of AMD (Figure 5e). There is an accompanying drop in the average radius of gyration by 2 Å, as the structure becomes more compact (Figure 5f). We also observe that the pyrin domain appears to more closely approach the LRR domain (maroon, Figure 5g), as the structure folds into what appears to be a more closed structure (Figure 5c,d). Clearly, the absence of NEK7 allows for a significant rearrangement of the LRR domain with respect to the NACHT domain.

### 3.3 Simulation of ATP‐bound NLRP3 model

For comparison, we substitute ATP for ADP within the cofactor binding pocket of the initial NEK7‐free NLRP3 model. In practice, the γ‐phosphate group was added to the bound ADP to form ATP (Section 2). Following the same approach as for the ADP‐bound NLRP3 simulation, we obtain a microsecond simulation of unbiased MD, followed by 600 ns of AMD. Interestingly, we observe a rather similar backbone RMSD profile as for the NLRP3 simulation with ADP cofactor, with high values for the NACHT and LRR domains indicating a relative reorientation (Figure S7b). In this case, however there appears to be a rotation in a different direction (green, Figure 5a,b), with a shift in mean dihedral angle from −33° to −2° over the last 600 ns of MD (Figure 5e); this latter value is on average maintained over the AMD simulation, with a value of 0° over the last 200 ns of AMD, although with large fluctuations (±23°). As this movement occurs, there also appears to be an opening up of the LRR‐NACHT angle (dark green, Figure 5c); however, there is only a modest increase in the radius of gyration over MD and AMD (Figure 5f). The distance between PYD and LRR domains also gradually increases, from 75 Å to a value of 90 Å by the end of the AMD trajectory (dark green, Figure 5g). This opening and change in pyrin orientation on ATP binding may be significant in terms of activation, with the requirement of pyrin–pyrin interactions in ASC polymerization.

**FIGURE 6:**
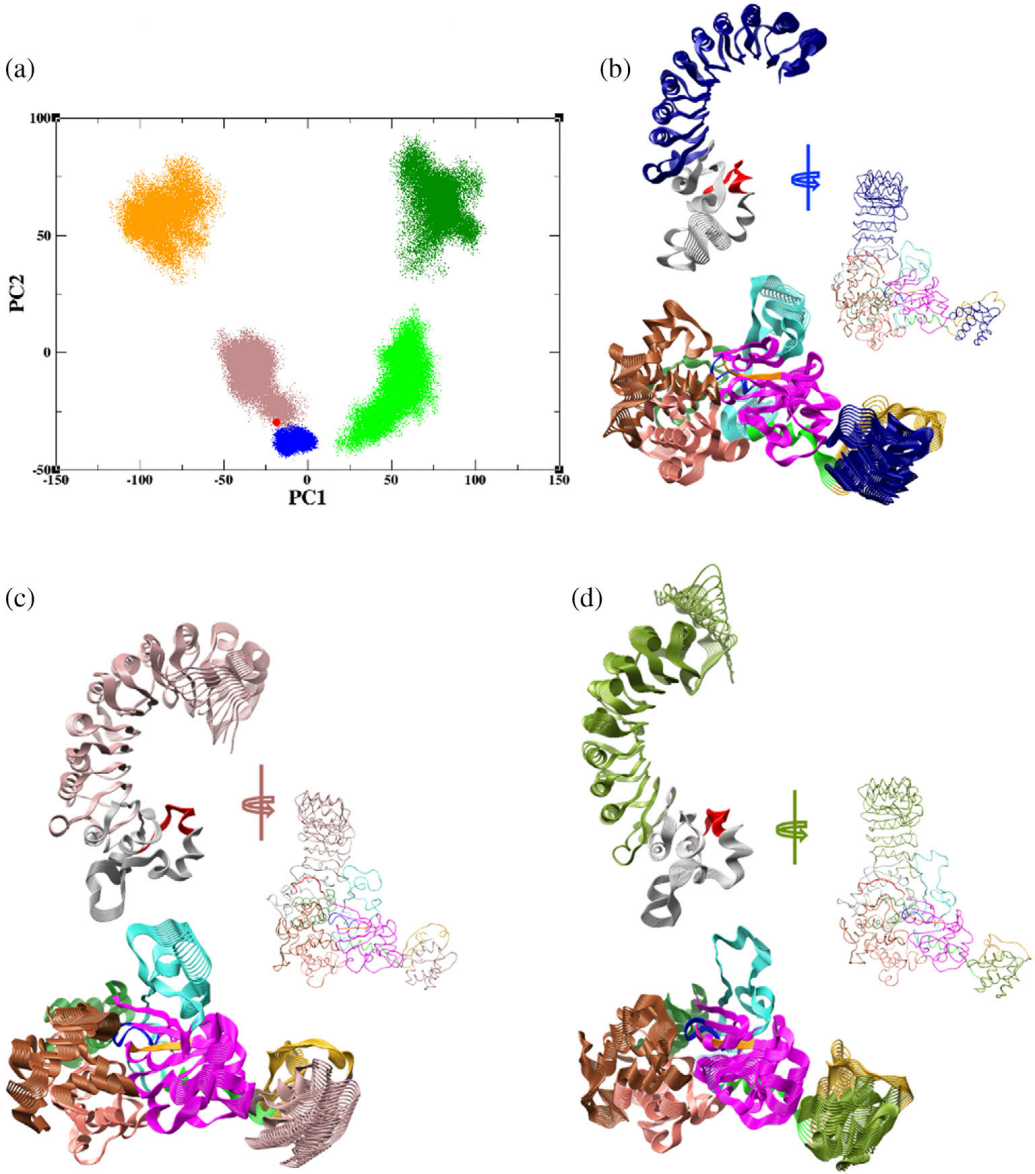
Principal component analysis of trajectories: (a) projection of the trajectories on the space defined by the two major principal components, PC1 and PC2, of ADP‐bound NLRP3/NEK7 (blue); of ADP‐bound NLRP3 from MD (brown) and AMD (orange); of ATP‐bound NLRP3 from MD (green) and AMD (dark green); and NLRP3/NEK7 cryo‐EM structure (red). Visualization of movement along principal eigenvectors from microsecond MD of (b) NLRP3/NEK7, (c) ADP‐bound NLRP3, and (d) ATP‐bound NLRP3. Note that the subdomains are colored using the same scheme as in Figure 1c. For clarity, LRR is shown separately

Complementing the simulated trajectories of NLRP3/NEK7, and ADP‐bound and ATP‐bound NLRP3 (Videos [Link], [Link] respectively), PCA of these dynamical trajectories provides a useful characterization of the distinct regions of phase space explored. For the microsecond MD of NLRP3–NEK7 complex, a limited region in principal component space proximal to the cryo‐EM structure is explored (blue and red, respectively, Figure 6a), reflecting the relative rigidity of NLRP3 due to the interactions with NEK7. In the absence of NEK7, the ADP‐bound NLRP3 molecule shifts to occupy a different and somewhat larger cluster over the last 500 ns of the MD simulation, corresponding to a different NACHT‐LRR orientation (brown, Figure 6a). On application of AMD, a further shift in conformation is observed, as well as a more dispersed cluster of conformations (orange, Figure 6a). A similar pattern is observed for the ATP‐bound NLRP3 simulations, with clusters of increasing size and distance from the cryo‐EM structure (red, Figure 6a) for MD (green, Figure 6a) and AMD trajectories (dark green, Figure 6a). However, the phase space occupied is distinct from that occupied by the ADP‐bound NLRP3 simulation.

For these simulations, the eigenvectors of the principal components, corresponding to the lowest frequency modes of motion, can be visualized (Figure 6b–d; see Videos [Link], [Link] for NLRP3/NEK7, ADP‐bound and ATP‐bound NLRP3 respectively); the modes indicate significant motion across the NLRP3 molecule, including the PYD domain, the C‐terminal end of the LRR domain and the FISNA motif. We also note motion in the secondary structural features of the NACHT and trLRR region which sit in the hinge region. Overall, these features correspond to a cooperative, almost piston‐like, motion across the NLRP3 molecule, where the NACHT and LRR open and close at this hinge in a potential activating movement; this is most readily visualized through the animation of the eigenvectors when the simulations of all three systems are combined (see Video S7).

### 3.4 NLRP3–cofactor interactions

**FIGURE 7:**
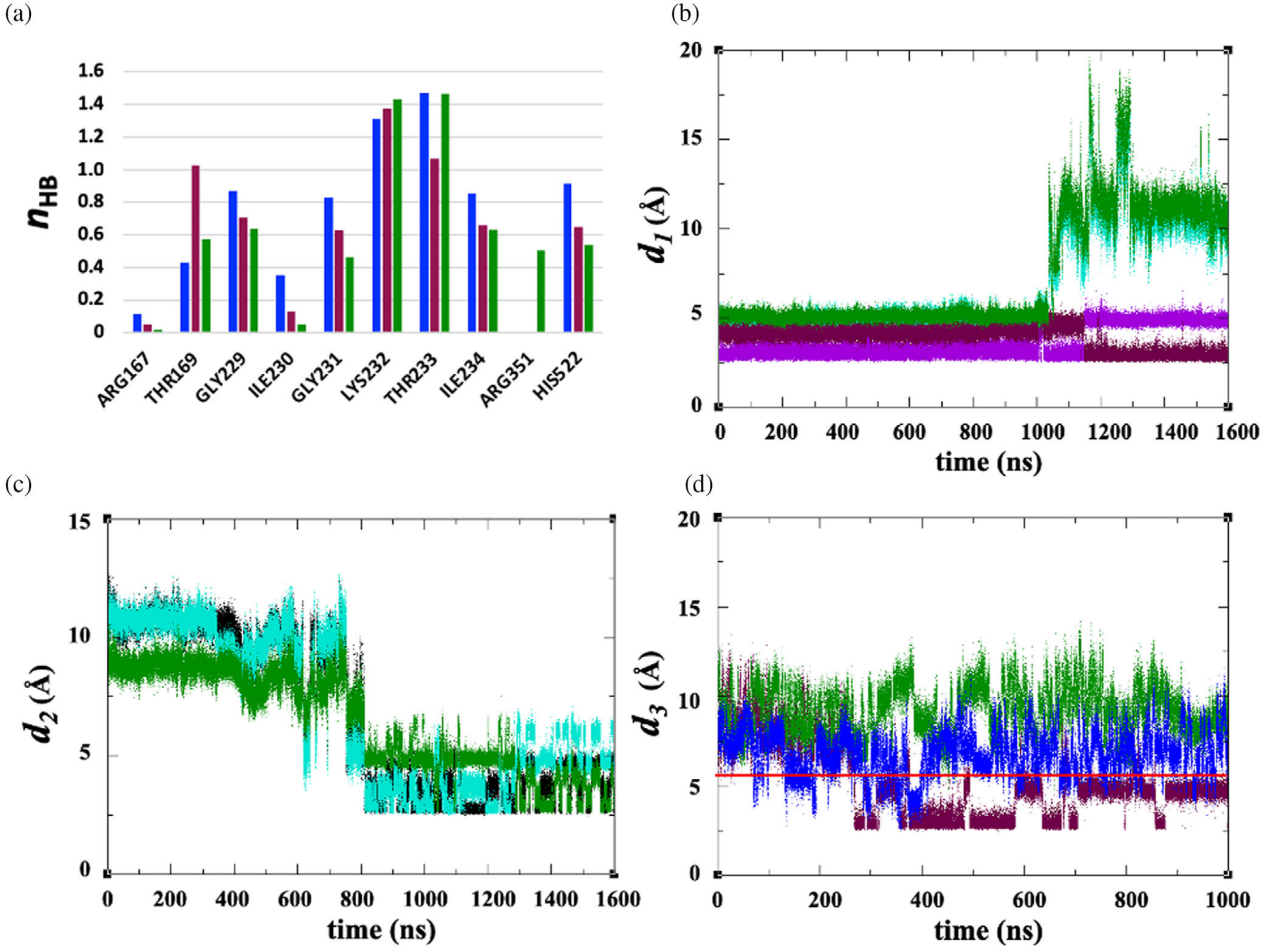
(a) Mean number of hydrogen bonds *n*_HB_ between cofactor and NLRP3 binding site, for MD simulation of NLRP3/NEK7 (blue), and MD/AMD simulation of ADP‐bound NLRP3 (maroon) and ATP‐bound NLRP3 (dark green). (b) Time series of distance *d*_1_ between His522 Nε_2_: and O1β atoms of ADP (maroon) or ATP (dark green); and O2β of ADP (purple) or ATP (cyan) over combined 1,600 ns MD/AMD simulation. (c) Time series of distance *d*_2_ between ATP O3γ and Arg351 atoms Nη_1_ (dark green), Nη_2_ (cyan), and Nε (black). (d) Time series of distance *d*_3_ between Glu527 Oε_1_ and Arg351 Nη_1_ over microsecond MD of NLRP3/NEK7 (blue), ADP‐bound NLRP3 (maroon), and ATP‐bound NLRP3 (dark green). The red line represents a value of 5.6 Å for distance *d*_3_ in the 6NPY cryo‐EM structure

To understand the origin of this difference in the dynamical opening–closing behavior of the ADP‐ and ATP‐bound NLRP3 simulations, we examine the interatomic interactions of the cofactor with its NLRP3 binding site over the MD and AMD simulations. Most of the interactions in both ADP‐ and ATP‐bound NLRP3 simulations reflect those found in the MD simulation of the NLRP3/NEK7 complex, when comparing the number of hydrogen bonds (Figure 7a) in the cofactor pocket (Figure S6 and Table S6). There appears to be a reduced number of hydrogen bonds from cofactor to Thr233 in the ADP‐bound NLRP3 simulation, with a drop from 1.5 to 1.1 interactions on average; however, there is a converse increase in interaction with Thr169, relative to ATP‐bound NLRP3, from 0.4 to 1.0 (Figure 7a). Interestingly, there is somewhat reduced interaction with His522 of the WHD subdomain of NACHT, for both the ADP‐bound and ATP‐bound NLRP3 simulations relative to the NLRP3/NEK7 complex, with values of 0.5, 0.6, and 0.9 interactions, respectively (Figure 7a).

This relative similarity in the population of cofactor‐His522 hydrogen bonds obscures the fact that the cofactor β‐phosphate distance *d*_*1*_ to His522 increases significantly in the ATP‐bound simulation from 5 to 12 Å (Figures 7b and S6f); this occurs at 1053 ns, shortly after application of AMD. For the ADP‐bound structure, however, the His522 remains in close proximity to the cofactor over MD and AMD (Figures 7b and S6e). This accords with a suggestion by Dekker et al.^14^ that His522 is important in maintaining the inactive closed conformation of NLRP3.

We also note the formation of a strong hydrogen bond by Arg351 with the γ‐phosphate of ATP (Figures 7a,c and S6d,f); no interaction with this residue is found in the ADP‐bound NLRP3 simulations. This Arg351 is part of the sensor 1 motif in the NBD, and is thought to play a role in coordinating ATP in NLRP3 and facilitating interdomain rearrangement, as well as in binding inhibitor MCC950.^14^, ^43^ This Arg351 residue makes a hydrogen bond with Glu527 in the cryo‐EM ADP‐bound NLRP3 structure (Figure 7d); this interaction is maintained in the MD simulation of this system and in the NEK7‐free system. However, the interaction is lost in the ATP‐bound NLRP3 simulation, as Arg351 instead forms the expected^15^ interaction with the γ‐phosphate of ATP (Figures 7a and S6f).

### 3.5 Interdomain interactions

**FIGURE 8:**
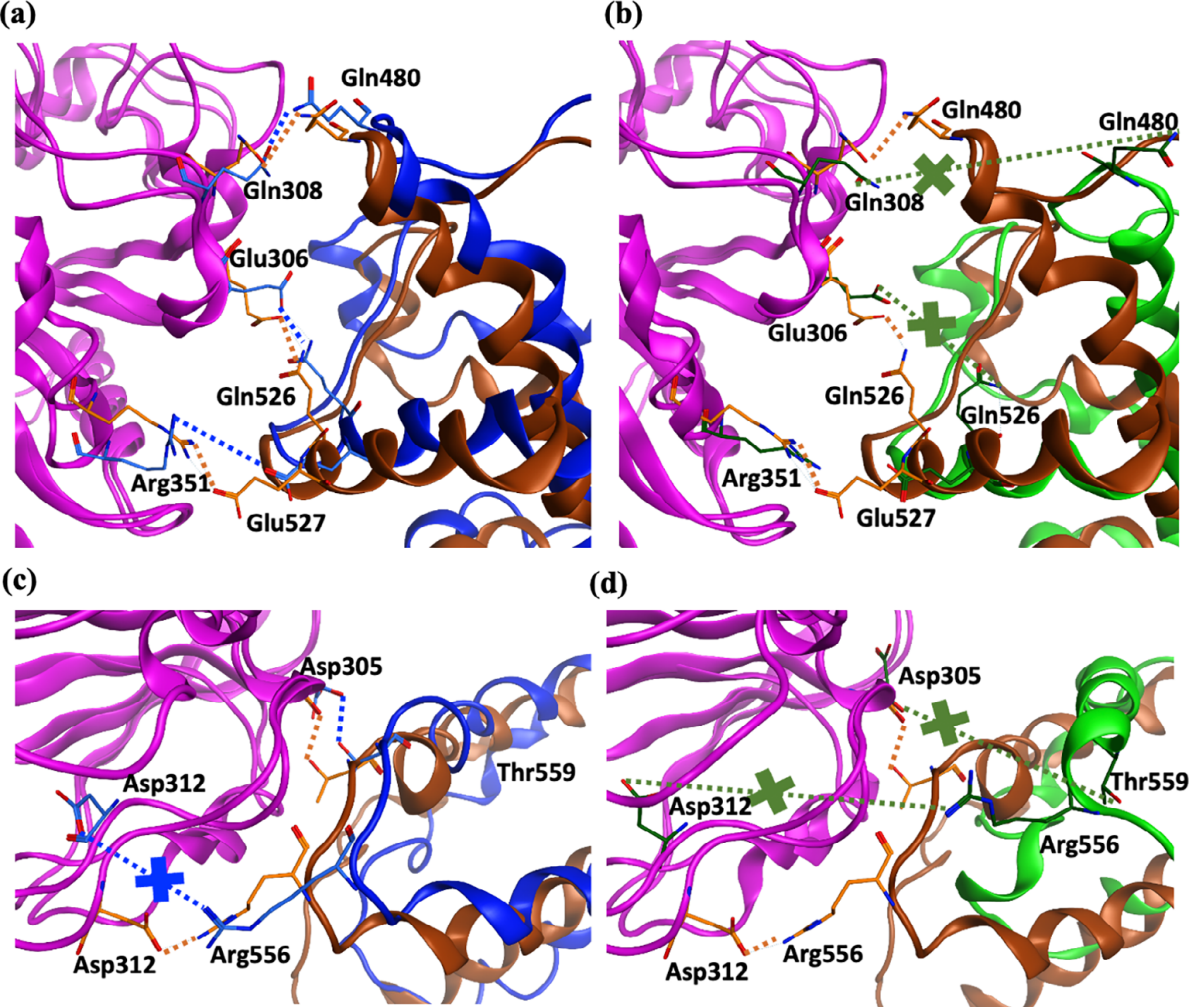
Selected hydrogen bond interactions at NBD/WHD interface for (a) ADP‐bound NLRP3/NEK7 (blue) and NLRP3 (brown) simulations; and for (b) ADP‐bound (brown) and ATP‐bound (green) NLRP3 simulations. Selected hydrogen bond interactions at NBD/HD2 interface for (c) NEK7 (blue) and ADP‐bound (brown) simulations and (d) ADP‐bound (brown) and ATP‐bound (green) simulations. In all cases, NBD domain in magenta; interactions lost between compared simulations indicated with a cross

Beyond the immediate changes in cofactor interactions, we may observe rearrangements in interdomain hydrogen bonding associated with the opening‐closing behavior of NLRP3. Interactions at the NBD/WHD interface of ADP‐bound NLRP3, in the presence or absence of NEK7, are rather similar (Figure 8a). For example, the hydrogen bond between Gln308 Oε1^…^Gln480 Nε2 spanning the NBD and WHD subdomain is maintained in both simulations (Figures 8a and S8a). However, when ATP‐bound, this hydrogen bond is lost in NLRP3 (Figures 8b and S8a). Indeed, there appears to be a significant repositioning of the interface, with the movement of helix α2 in the WHD (Figure 8b).

At the NBD‐HD2 interface, the interaction indicated by the distance Asp305 O^…^Oγ_1_ Thr559 is tightly formed in the ADP‐bound NLRP3 and NLRP3/NEK7 simulations (Figure 8c, S8b) but is lost in the ATP‐bound NLRP3 simulation (Figure 8d, S8b). We also note the loss of the NBD‐HD2 salt bridge between Asp312 Oδ_2_^…^ Nη_1_ Arg556 in the ATP‐bound simulation (Figure 8d and S8c). These changes in interaction reflect a conformational rearrangement of the NLRP3 molecule stemming from the additional phosphate group of the cofactor.

### 3.6 Conformation of the acidic loop of NLRP3

**FIGURE 9:**
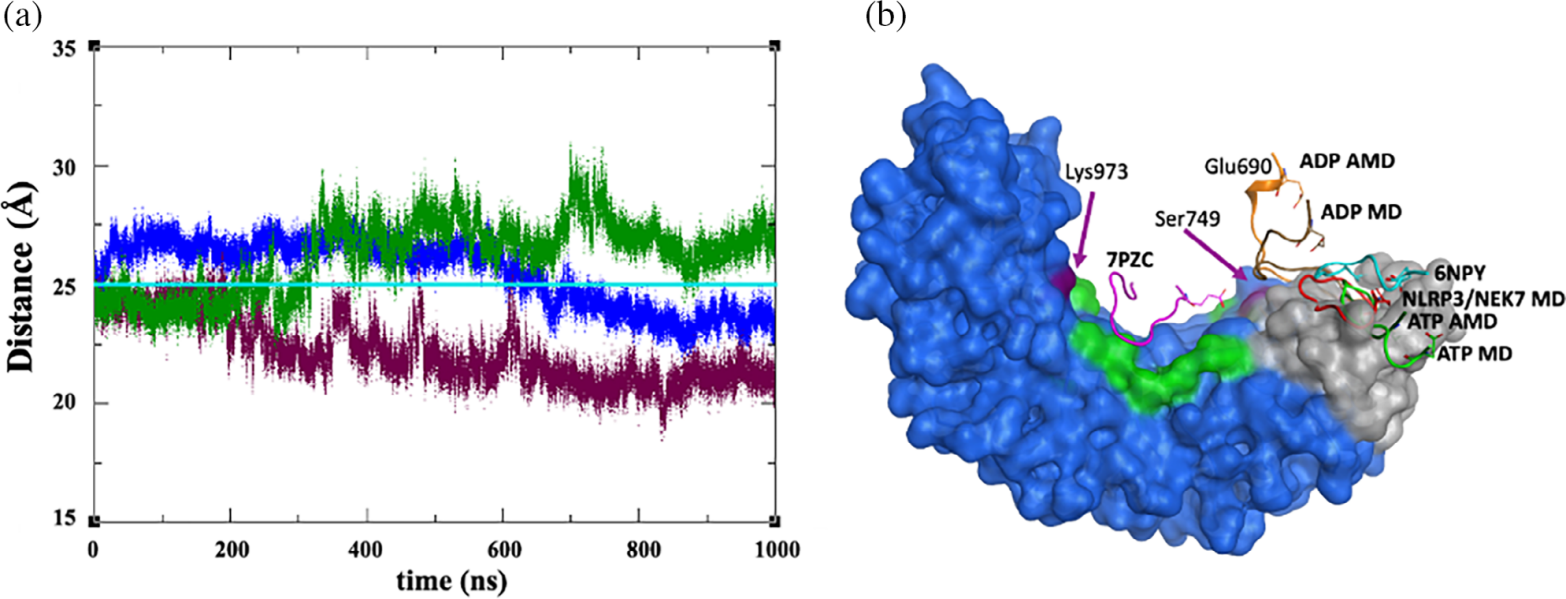
(a) Time series of the distance between the acidic loop and LRR, indicated using C_α_ atoms of Glu690 and Ser749, respectively, over microsecond MD of ADP‐bound NLRP3/NEK7 (blue), ADP‐bound NLRP3 (maroon) and ATP‐bound NLRP3 (dark green). The cyan colored line represents the 6NPY cryo‐EM value. (b) Acidic loop conformations from the final configuration of ADP‐bound NLRP3/NEK7 (red); of ADP‐bound NLRP3 from MD (brown) and AMD (orange); of ATP‐bound NLRP3 from MD (green) and AMD (dark green); NLRP3/NEK7 cryo‐EM structure 6NPY (cyan); and cryo‐EM structure of inactive NLRP3 oligomer^13^; (magenta); surface of trLRR (gray), cLRR (blue) and the basic patches of cLRR (green) of 6NPY also shown. The surface of the first (Ser749) and the last (Lys973) residues of the basic patch is colored purple

As well as evaluating the change in overall conformation of the NLRP3 as a function of NEK7 and cofactor, we consider more localized features. In particular, we consider the behavior of the acidic loop of the trLRR subdomain. This loop remains in its cryo‐EM conformation in the presence of NEK7 over the microsecond MD simulation (Figure 9a). Once NEK7 is removed, the loop remains in this location and does not sample in the direction of the basic region of LRR identified by Hochheiser et al.^13^ in their cryo‐EM cage structure of MCC950‐bound inactive NLRP3 (Figure 9b); this is also true in the ATP‐bound form over the microsecond simulation (dark green, Figure 9a).

On the application of AMD to both ADP‐bound and ATP‐bound NLRP3 simulations, the loop in ADP‐bound NLRP3 appears to explore a wider range of conformations (Figure 9b) but does not form interactions with this basic region on the timescale of the simulation. This suggests either further sampling is required, that the interaction is weak or that the conformation observed arises due to the presence of the inhibitor MCC950 in the cryo‐EM structure, trapping the molecule in an inactive conformation and the acidic loop in this particular interaction. We note that in other inactive cryo‐EM structures, the acidic loop conformation is unresolved.

### 3.7 Cofactor pocket accessibility

**FIGURE 10:**
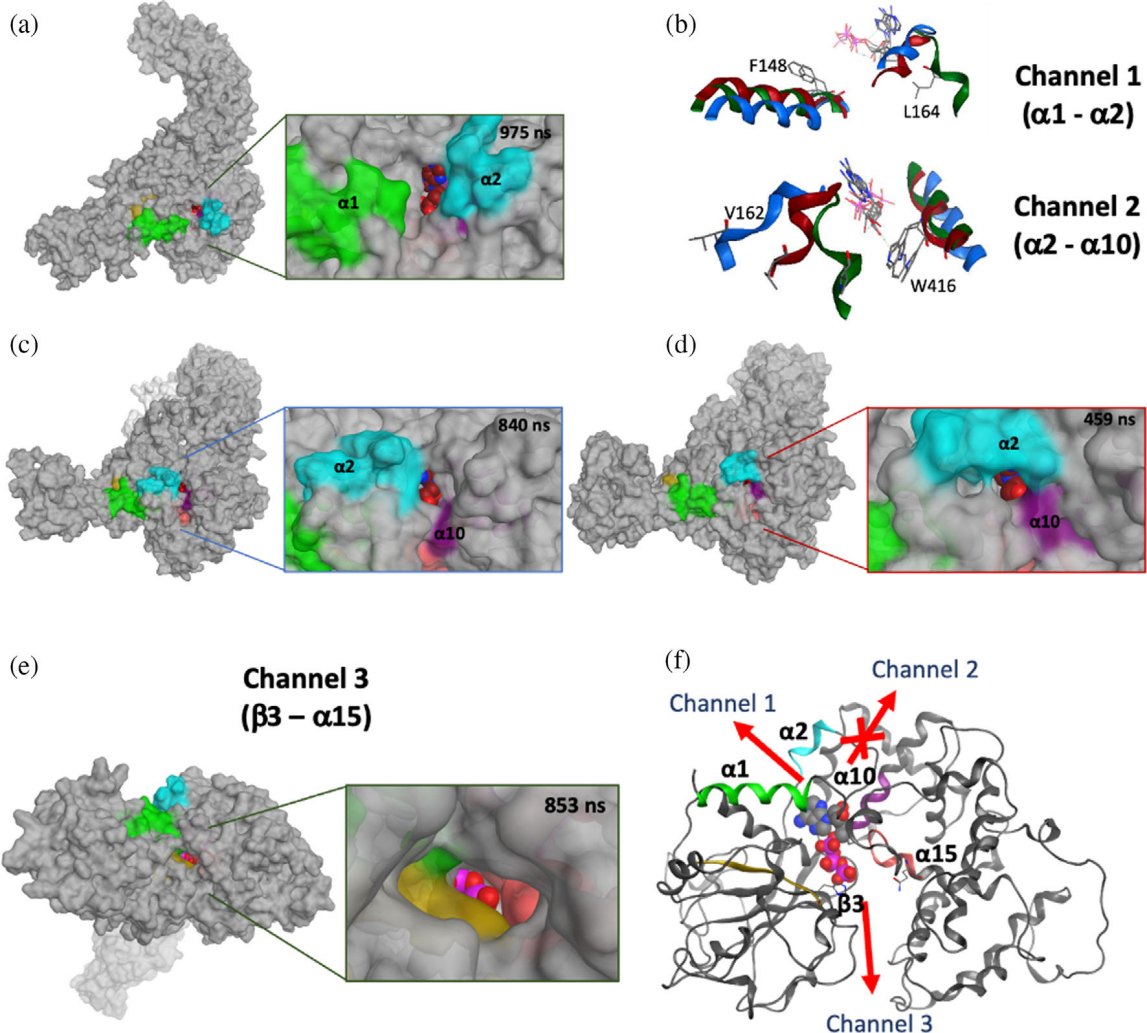
Cofactor binding site accessibility. (a) Side‐view of ATP site accessibility, between α1 (green) and α2 (cyan) helices of NACHT domain in ATP‐bound NLRP3 simulation using the structure at 975 ns showing the opening of channel between helices α1 and α2 of NACHT domain and exposure of cofactor. (b) Comparison of site accessibility of ATP‐bound NLRP3 (dark green), NEK7‐bound NLRP3 (blue) and ADP‐bound NLRP3 (maroon). ADP accessibility, between α2 (cyan) and α10 (purple) helices of NACHT domain in (c) NEK7‐bound NLRP3 simulation using the structure at 840 ns and (d) ADP‐bound NLRP3 simulation using the structure at 459 ns. (e) ATP accessibility “top‐view” between the third β‐sheet (gold) and sensor 2‐like motif (pink) of NACHT domain using the structure at 853 ns. (f) Ribbon representation of NACHT domain using the structure of ATP‐bound simulation at 853 ns showing Channels 1, 2, and 3. Cofactor colored by atom type (P; magenta, O; red, C; gray and N; blue) and the channel inaccessibility is indicated by cross

Finally, we consider the accessibility of the cofactor pocket. From an analysis by Tapia‐Abellán et al.,^11^ it was previously observed that this pocket appeared inaccessible from the protein surface in the NEK7‐bound cryo‐EM structure.^9^ However, their modeled structure of the fully open NLRP3 geometry, based on analogy with NLRC4, indicated a potential channel between helices α1 and α2 of the FISNA region of the NACHT domain.^11^ Correspondingly, in our simulations of NEK7‐bound or NEK7‐free NLRP3/ADP here, we similarly do not see an obvious point of entry for the cofactor between these two helices (Figure S9). We may describe this putative channel as Channel 1, defined by C_α_–C_α_ interatomic distance, denoted *d*_1_, between Phe148 and Leu164. For the ADP‐bound microsecond trajectories of NLRP3, with or without NEK7 bound, values of *d*_1_ of only ~10 Å are found (Figure S10). However, for the ATP‐bound NLRP3 simulation, this distance fluctuates up to 16 Å (Figure S10). This increased accessibility appears to be due to the shift of the α2 helix in this system (Figures 10a and S10).

Although Channel 1 was not accessible in the ADP‐bound NLRP3 simulations, a related avenue, Channel 2, appears to be accessible for these simulations on the distal side of α2, between this helix and α10 (HD1) of the NACHT domain (Figure 10b). We describe Channel 2 using the Val162 C_α_–Trp416 C_α_ interatomic distance *d*_2_. This distance reaches an average value of 20 Å over the last 200 ns of the microsecond NEK7/NLRP3 simulation (Figures 10c and S10). This channel was somewhat smaller in the NEK7‐free microsecond MD simulations, with maximum distances *d*_*2*_ of 17 and 14 Å for ADP‐bound and ATP‐bound NLRP3, respectively (Figure S10 and 10d).

Another possible route of cofactor exchange, Channel 3, is observed in the ATP‐bound MD simulation. This is near‐orthogonal to Channels 1 and 2, and lies between β3 and the sensor 2‐like motif (residues 520 to 530) of the NACHT domain (Figure 10e). Channel 3 can be characterized by the distance *d*_*3*_ between C_α_ atoms of His260 and Gln526; this distance gradually increased from an initial value of 17 Å in the cryo‐EM structure, to ~19 Å in the ATP‐bound MD simulation (Figure S10), and then to 22 Å via AMD simulation.

**FIGURE 11:**
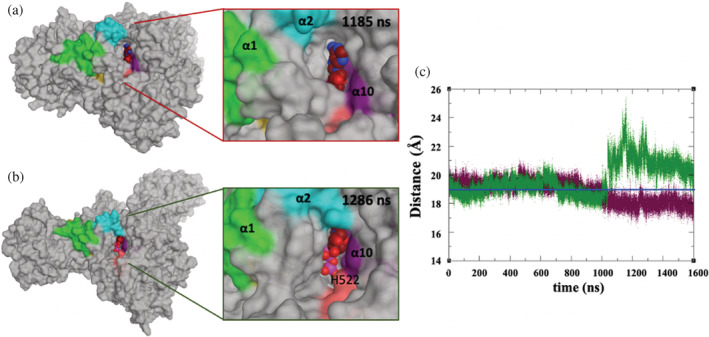
Cofactor pocket accessibility during MD/AMD simulation of (a) ADP‐bound NLRP3 model at 1185 ns and (b) ATP‐bound NLRP3 model at 1286 ns, showing that His522 is exposed in the ATP‐bound model while buried in the ADP‐bound model; this is also indicated by (c) time series of Thr169‐His522 C_α_–C_α_ distance of ADP‐bound NLRP3 (maroon) and ATP‐bound NLRP3 (dark green); blue line is an average value from last 500 ns trajectory of NEK7/NLRP3 simulation

On application of AMD simulations, Channel 1 does not appear to open wider than observed in the unbiased MD simulations of the NLRP3 systems (data not shown). However, there is an increased opening of Channel 2 during the application of AMD to the ADP‐bound NLRP3 model, to a distance of ~20 Å (Figure 11a). Similarly, during AMD of the ATP‐bound NLRP3 model, we note the further opening of Channel 3, to the extent that the sensor 2 residue, His522, is exposed (Figure 11b): the His522 C_α_–C_α_ distance to Thr169 of the FISNA region increases considerably when AMD is applied, to a value of ~22 Å (Figure 11c). This opening corresponds to the loss of the hydrogen bonds made by these residues with the ATP cofactor. These channels, for both ADP and ATP systems, provide indications of possible routes of cofactor access; and indicate their potential to more fully open given a suitable mechanical or chemical stimulus. It is interesting to consider the accessibility of the cofactor pocket in the context of the inactive cage structure. To this end, we took the conformation of NLRP3 from the NEK7‐bound simulation which displayed an open Channel 2 (Figure 10c) and modeled it onto the orientation of the monomers in the cage assembly structure of Hochheiser et al.^13^ (Figure S11a–d). It was evident that this channel would remain accessible within this quaternary framework. In fact, Channel 2 is present, albeit to a lesser degree, in the NLRP3 cage cryo‐EM structure of Hochheiser et al.^13^ itself (Figure S11d,e).

## 4 CONCLUSIONS

We have constructed and analyzed dynamical computational models of full‐length NLRP3 in the presence and absence of NEK7 interactions. NEK7 forms effective contacts with several NLRP3 domains and results in a rather rigid complex. These NEK7–LRR interactions are numerous, appear of good affinity, and may be able to compete with the LRR–LRR interactions observed in inactive oligomer forms of NLRP3. It is thought that the membrane‐bound double ring cage of inactive NLRP3 is in equilibrium with its monomer and dimer forms: this has been estimated from sucrose gradient profiles and representative negative‐staining EM images as 11% free and 89% membrane‐bound.^10^ The pyrins are localized and shielded by the cage; however, the polybasic regions may be solvent exposed as sensors to allow access when the potassium concentration falls intracellularly. Based on our simulation of ADP‐bound NLRP3, we propose that in the free monomeric form (when not in interphase), the NLRP3 molecule folds into a more compact shape than found in the NLRP3 inactive cage; the latter is near‐identical to the NLRP3/NEK7 bound form regarding its NACHT–LRR conformation.

However, when the additional γ‐phosphate of the ATP is present, our microsecond MD and 600 ns AMD simulations observe a change in conformation, with an opening of the NACHT‐LRR angle and shift in the presentation of the pyrin domain relative to ADP‐bound forms. These changes also involve the movement of subdomains within NACHT and are linked with shifts in hydrogen bonding between residues and with the cofactor. We note that short 10 ns simulations^18^ found structural differences in NACHT‐LRR orientation for ADP versus ATP‐bound NLRP3, although these differ in the details of the transition.

Our simulations indicate that cooperative motions within NLRP3 permit a NACHT‐LRR hinge movement, with closing and opening of these domains relative to one another as a function of ADP or ATP binding. Indeed, PCA indicates the discrete pathway of these changes in conformation as a function of the cofactor, with the NEK7‐bound cryo‐EM structure appearing to represent a partially closed semi‐active form (as suggested elsewhere^11^). Even after AMD, however, the ATP‐bound NLRP3 does not adopt the completely opened form that would be anticipated for the single ring disc oligomer (Figure 1a), analogous to the active conformation of NLRC4.^8^ It may be that other aspects of the activation mechanism need considering to capture this, or that there is a different pathway altogether from NLRC4 or that further conformational sampling is required. Nevertheless, these simulations indicate low‐frequency modes of coupled (sub)domain motion along which such an opening‐closing movement would likely travel, and provide insights into potential modulation of these dynamics by cofactor and subdomain interactions.

During these MD and AMD simulations of NLRP3, we note that we do not see an association of the trLRR acidic loop with the basic cluster of residues found in the cLRR at any point. It appears that this may be a feature of the specific cryo‐EM inactive cage structure, as it is not observed in other inactive oligomer NLRP3 cryo‐EM structures. Finally, we note that although the NEK7‐bound cryo‐EM structure of NLRP3 does not immediately suggest an access channel to the cofactor site, the MD and AMD simulations here indicate some plasticity in NLRP3 topology, with potential channel openings found for both the ADP‐bound and ATP‐bound NLRP3 systems (Figure 10f). In particular, a channel appears to be evident and accessible in the monomer and in the inactive cage oligomer forms.

While the complete mechanism of activation of the NLRP3 inflammasome remains to be elucidated, the interaction with NEK7 does appear to stabilize a more open, active conformation, prior to the full mechanochemical activation consequent on ATP binding and/or hydrolysis. It is becoming increasingly clear that NLRP3 aggregate plays a key role in the inactive as well as active forms, including in membrane‐mediated transport to its site of action. However, the transition from inactive double ring cage to active single ring disc is as yet unclear. No doubt NEK7 plays a role, but further work is required to determine whether disassembly to monomeric NLRP3, an *in situ* reorganization of the oligomer or another pathway occurs. As our understanding of the mechanism of NLRP3 progressively advances, with its associated conformational and dynamical intricacies, further opportunities to develop therapeutics targeting the inflammasome will emerge.

## AUTHOR CONTRIBUTIONS

**Sherihan El‐Sayed:** Formal analysis (equal); investigation (equal); methodology (equal); writing – original draft (equal); writing – review and editing (equal). **Sally Freeman:** Conceptualization (equal); investigation (equal); supervision (equal); writing – review and editing (equal). **Richard Bryce:** Conceptualization (equal); formal analysis (equal); funding acquisition (equal); methodology (equal); supervision (equal); writing – original draft (equal); writing – review and editing (equal).

## Supporting information


**FIGURE S1** Ramachandran plot of both cryo‐EM (a) and NLRP3 NACHT‐LRR model (b) using RAMPAGE online server (http://mordred.bioc.cam.ac.uk/~rapper/rampage.php), which revealed that 86.6 and 87.3% of residues of the model and the cryo‐EM fall in the favored region, respectively
**Figure S2** ProSA analysis results of the cryo‐EM and NLRP3 NACHT‐LRR model. Overall model quality of cryo‐EM (a) and NLRP3 NACHT‐LRR (b) which shows a fair model quality in terms of its overall energy compared to other solved PDB protein structures of similar size. Local model quality of 6NPY cryo‐EM structure (c) and NLRP3 NACHT‐LRR (d) in which the Z‐score of the NLRP3 NACHT‐LRR model (−7.5) is better than for the cryo‐EM (−6.6) which means that the overall model quality compared to other PDB structures is improved.
**Figure S3** Verify 3D scores for (a) the NLRP3 NACHT‐LRR model shows that 72% of the residues have averaged 3D‐1D scores of more than 0.2, as compared to 66% for (b) the 6NPY cryo‐EM structure.
**Figure S4** Secondary structure analysis of the initial model of NLRP3/NEK7 model (a) restrained over 50 ns and (b) 1,000 ns production MD, for residues 1–1,036. Secondary structure analysis of the initial model of NEK7/NLRP3 model restrained over 50 ns of MD simulation for (c) NEK7, (d) PYD, (e) NACHT, and (f) LRR; compared to the structure over 1,000 ns production for (g) NEK7, (h) PYD, (i) NACHT and (j) LRR. The color code in all plots is black (parallel β‐sheets), red (anti‐parallel β‐sheets), green (3–10 helices), blue (α helices), yellow (π helices), brown (turn), and gray (bend).
**Figure S5** Structure of LRR domain β‐sheets: cryo‐EM structure (red); NLRP3/NEK7 structure at 700 ns of microsecond MD simulation (purple).
**Figure S6** Cofactor interactions of NLRP3 for (a) the 6NPY cryo‐EM; for MD structure after 1 μs for (b) ADP/NLRP3/NEK7; (c) ADP/NLRP3; (d) ATP/NLRP3; and after AMD simulation for (e) ADP/NLRP3 and (f) ATP/NLRP3.
**Figure S7** Time series of backbone RMSD for PYD (red), NACHT (purple), and LRR (cyan) over a combined 1,600 ns MD/AMD simulation of (a) ADP‐bound NLRP3 and (b) ATP‐bound NLRP3.
**Figure S8** Time series of interatomic distances between (a) Gln308 Oε1(from NBD) and Gln480 Nε2 (from WHD), denoted d1; (b) Asp305 O (from NBD) – Thr559 Oγ1 (from HD2) distance, denoted d2; and (c) Asp312 Oδ2 (from NBD) – Arg556 Nη1 (from HD2) distance, denoted d3; for simulation of NLRP3/NEK7 (blue), ADP‐bound NLRP3 (maroon) and ATP‐bound NLRP3 (dark green). In all cases, the red line represents the distance value in the cryo‐EM for d1 (4.6 Å) and in the initial model for d2 (7.14 Å) and d3 (13.7 Å). Note that the residue Thr559 and the side chain of Arg556 are unresolved in the 6NPY cryo‐EM structure.
**Figure S9** Cofactor accessibility “side‐view” between α1 (green) and α2 (cyan) helices of NACHT domain. NLRP3/ADP (1 μs) NLRP3/ATP (1 μs) Initial model NLRP3/NEK7 (700 ns)
**Figure S10** (a) Cofactor accessibility between α1 (green) ‐ α2 (cyan) helices of NACHT domain for ATP/NLRP3 simulation; and between α2 (cyan) ‐ α10 (purple) helices of NACHT domain for ADP/NLRP3 simulation. In all cases, the cofactor inaccessibility is indicated with a cross. Time series of (b) distance d1 Phe148 – Leu164 Cα‐Cα, (c) distance d2 Val162 – Trp416 Cα‐Cα and (d) distance d3 His260 Cα – Gln526 Cα distances for the microsecond simulation of NLRP3/NEK7 (blue), ADP‐bound NLRP3 (maroon) and ATP‐bound NLRP3 (dark green). In all cases, the red line represents the distance value in the cryo‐EM for d1 (8.7 Å), d3 (16.8 Å), and in the initial model for d2 (7.2 Å). Note that the residue Val162 is unresolved in the cryo‐EM.
**Figure S11** Cofactor accessibility in NLRP3 cage structure. Surface and ribbon representation of the modeled NLRP3 cage structure using the structure at 840 ns of NEK7‐bound NLRP3 simulation after deleting NEK7, PYD, and PYD‐NACHT linker; top view (a,b) and side view (c) showing the cofactor accessibility through channel 2. Cryo‐EM structure of inactive NLRP3 oligomer (PDB code 7PZC); top view (d) and side view (e).
**Table S1** AMD parameters for boosting of total potential energy (αtot, Etot) and dihedral energy terms (αdih, Edih). Values in kcal/mol.
**Table S2** Average values of backbone RMSD of NLRP3/NEK7 model (in Å) over microsecond MD simulation, with respect to using the initial input structure (labeled “initial”) or using the equilibrated structure (labeled “equil”). Standard deviation also shown.
**Table S3** Selected average distances (in Å) between NEK7 and LRR residues for 1,000 ns MD trajectory and 6NPY cryo‐EM structure. Standard deviation also shown.
**Table S4** Selected average distances (in Å) between NEK7 and HD2 residues for 1,000 ns MD trajectory and 6NPY cryo‐EM structure. Standard deviation also shown.
**Table S5** Selected average distances (in Å) between NEK7 and NBD residues for 1,000 ns MD trajectory and 6NPY cryo‐EM structure. Standard deviation also shown.
**Table S6** Selected average interatomic distances (in Å) between cofactor (ADP / ATP) and NLRP3 binding site residues from simulation of NLRP3/NEK7, ADP‐bound, and ATP‐bound NLRP3 over 1,000 ns MD simulation and for 6NPY cryo‐EM structure. Standard deviation also shown.
**Table S7** Average values of RMSD (in Å) of ADP‐bound and ATP‐bound NLRP3 from 1,000 ns MD and further 600 ns AMD. RMSD was calculated using as a reference either the initial input structure (i.e., NLRP3/NEK7 at 700 ns, labeled “initial”) or using the equilibrated structure (i.e., after 50 ns of equilibration of the NEK7‐free system, labeled “equil”). Standard deviation also shown.

Video S1

Video S2

Video S3

Video S4

Video S5

Video S6

Video S7

## Data Availability

The data that support the findings of this study are available from the corresponding author upon reasonable request.
