# Supplementary material for: Probing the effect of NEK7 and cofactor interactions on dynamics of NLRP3 monomer using molecular simulation
Source: Protein Sci. 2022 Sep 21;31(10):e4420. doi: 10.1002/pro.4420 (PMC9601872; doi:10.1002/pro.4420)
Supplement: Supplementary file 1 — FIGURE S1 Ramachandran plot of both cryo‐EM (a) and NLRP3 NACHT‐LRR model (b) using RAMPAGE online server (http://mordred.bioc.cam.ac.uk/~rapper/rampage.php), which revealed that 86.6 and 87.3% of residues of the model and the cryo‐EM fall in the favored region, respectively Figure S2 ProSA analysis results of the cryo‐EM and NLRP3 NACHT‐LRR model. Overall model quality of cryo‐EM (a) and NLRP3 NACHT‐LRR (b) which shows a fair model quality in terms of its overall energy compared to other solved PDB protein structures of similar size. Local model quality of 6NPY cryo‐EM structure (c) and NLRP3 NACHT‐LRR (d) in which the Z‐score of the NLRP3 NACHT‐LRR model (−7.5) is better than for the cryo‐EM (−6.6) which means that the overall model quality compared to other PDB structures is improved. Figure S3 Verify 3D scores for (a) the NLRP3 NACHT‐LRR model shows that 72% of the residues have averaged 3D‐1D scores of more than 0.2, as compared to 66% for (b) the 6NPY cryo‐EM structure. Figure S4 Secondary structure analysis of the initial model of NLRP3/NEK7 model (a) restrained over 50 ns and (b) 1,000 ns production MD, for residues 1–1,036. Secondary structure analysis of the initial model of NEK7/NLRP3 model restrained over 50 ns of MD simulation for (c) NEK7, (d) PYD, (e) NACHT, and (f) LRR; compared to the structure over 1,000 ns production for (g) NEK7, (h) PYD, (i) NACHT and (j) LRR. The color code in all plots is black (parallel β‐sheets), red (anti‐parallel β‐sheets), green (3–10 helices), blue (α helices), yellow (π helices), brown (turn), and gray (bend). Figure S5 Structure of LRR domain β‐sheets: cryo‐EM structure (red); NLRP3/NEK7 structure at 700 ns of microsecond MD simulation (purple). Figure S6 Cofactor interactions of NLRP3 for (a) the 6NPY cryo‐EM; for MD structure after 1 μs for (b) ADP/NLRP3/NEK7; (c) ADP/NLRP3; (d) ATP/NLRP3; and after AMD simulation for (e) ADP/NLRP3 and (f) ATP/NLRP3. Figure S7 Time series of backbone RMSD for PYD (red), NACHT (purple [file PRO-31-e4420-s001.pdf]

Supporting information

Probing the effect of NEK7 and cofactor interactions on dynamics of NLRP3 monomer using molecular simulation

*Sherihan El-Sayed,[1,2] Sally Freeman[1] and Richard A. Bryce[1]**

1 Division of Pharmacy and Optometry, School of Health Sciences, Manchester Academic Health Sciences Centre, University of Manchester, Oxford Road, M13 9PT, UK.

2 Department of Medicinal Chemistry, Faculty of Pharmacy, Zagazig University, Zagazig 44519, Egypt.

Figure S1 Ramachandran plot of both cryo-EM (a) and NLRP3$^{NACHT-LRR}$ model (b) using RAMPAGE online server (http://mordred.bioc.cam.ac.uk/~rapper/rampage.php) which revealed that 86.6% and 87.3% of residues of the model and the cryo-EM fall in the favored region, respectively.

(a) (b)

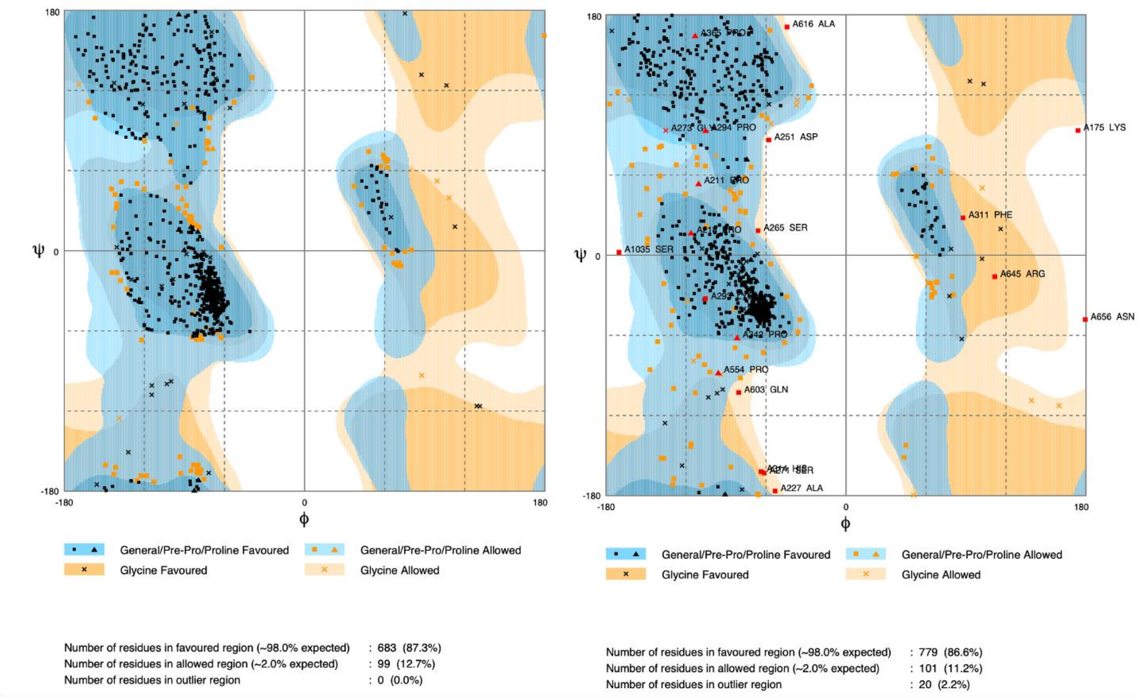

Figure S2 ProSA analysis results of the cryo-EM and NLRP3$^{\text{NACHT-LRR}}$ model. Overall model quality of cryo-EM (a) and NLRP3$^{\text{NACHT-LRR}}$ (b) which shows a fair model quality in terms of its overall energy compared to other solved PDB protein structures with similar size. Local model quality of 6NPY cryo-EM structure (c) and NLRP3$^{\text{NACHT-LRR}}$ (d) in which the Z-score of the NLRP3$^{\text{NACHT-LRR}}$ model (-7.5) is better than for the cryo-EM (-6.6) which means that the overall model quality compared to other PDB structures is improved.

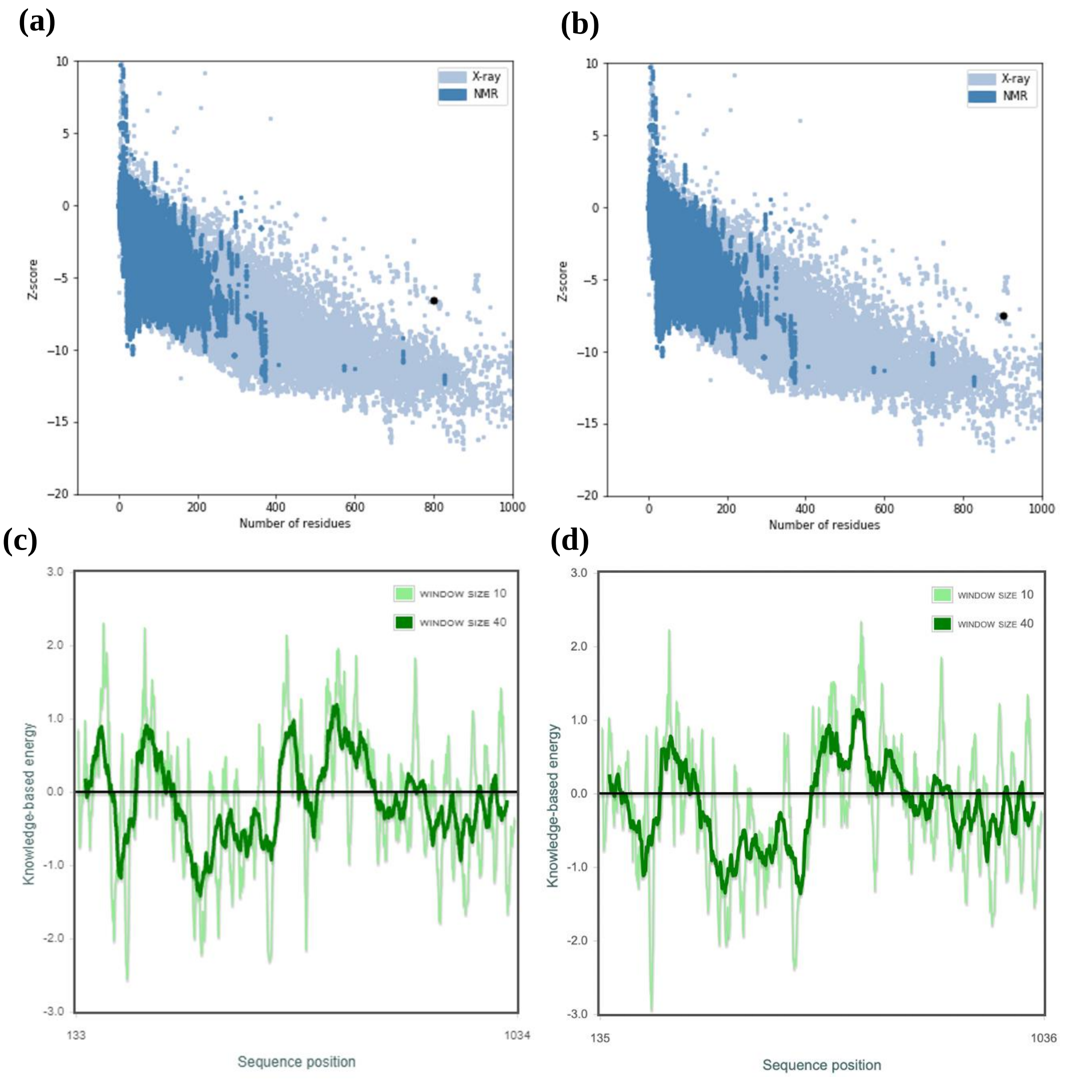

Figure S3 Verify 3D scores for (a) the NLRP3$^{NACHT\text{-}LRR}$ model shows that 72% of the residues have averaged 3D-1D scores of more than 0.2, as compared to 66% for (b) the 6NPY cryo-EM structure.

(a)

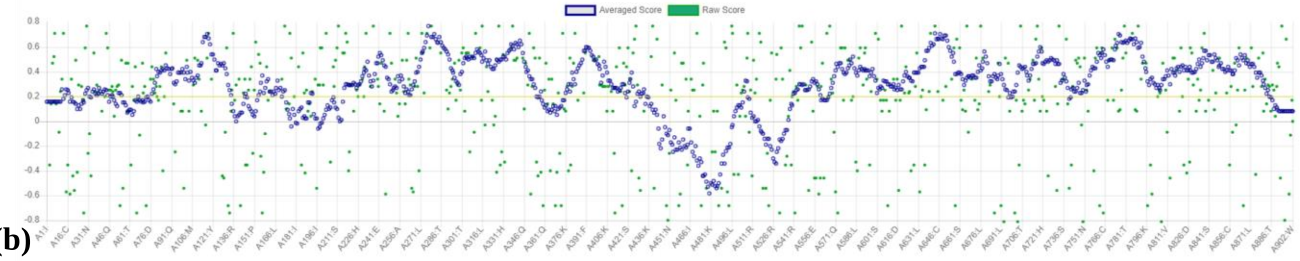

(b)

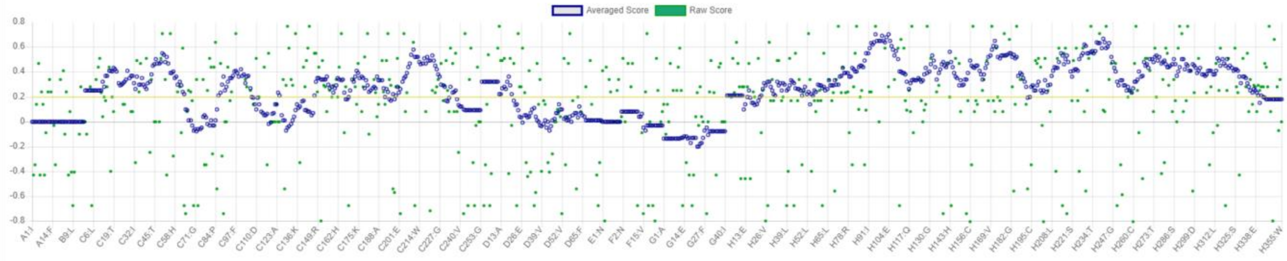

Figure S4 Secondary structure analysis of the initial model of NLRP3/NEK7 model (a) restrained over 50 ns and (b) 1000 ns production MD, for residues 1 - 1036. Secondary structure analysis of the initial model of NEK7/NLRP3 model restrained over 50 ns of MD simulation for (c) NEK7, (d) PYD, (e) NACHT and (f) LRR; compared to the structure over 1000 ns production for (g) NEK7, (h) PYD, (i) NACHT and (j) LRR. The colour code in all plots is black (parallel β-sheets), red (anti-parallel β-sheets), green (3-10 helices), blue (α helices), yellow (π helices), brown (turn) and grey (bend).

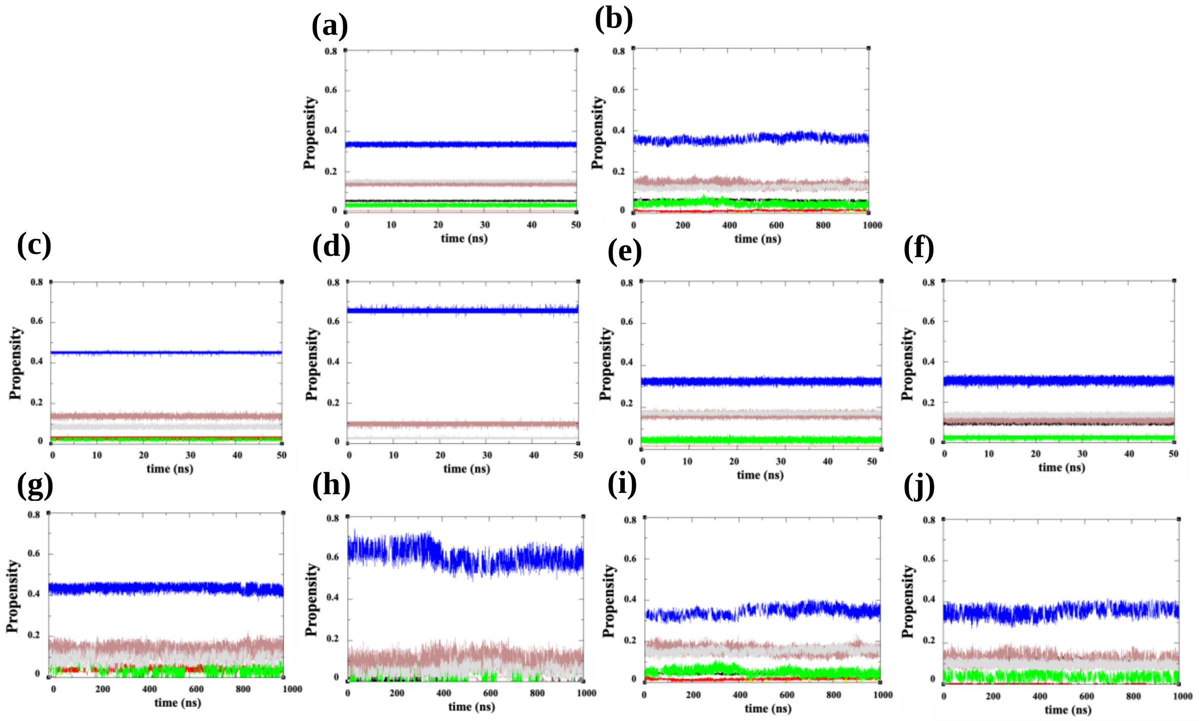

Figure S5 Structure of LRR domain β-sheets: cryo-EM structure (red); NLRP3/NEK7 structure at 700 ns of microsecond MD simulation (purple).

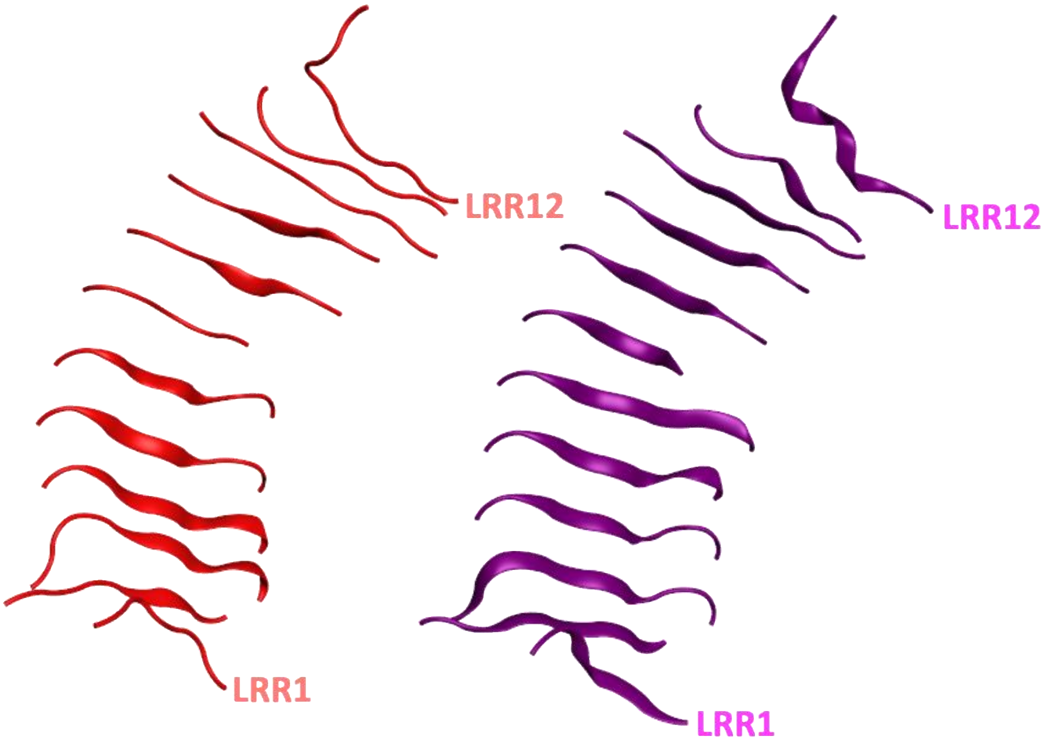

Figure S6 Cofactor interactions of NLRP3 for (a) the 6NPY cryo-EM; for MD structure after 1 μs for (b) ADP/NLRP3/NEK7; (c) ADP/NLRP3; (d) ATP/NLRP3; and after AMD simulation for (e) ADP/NLRP3 and (f) ATP/NLRP3.

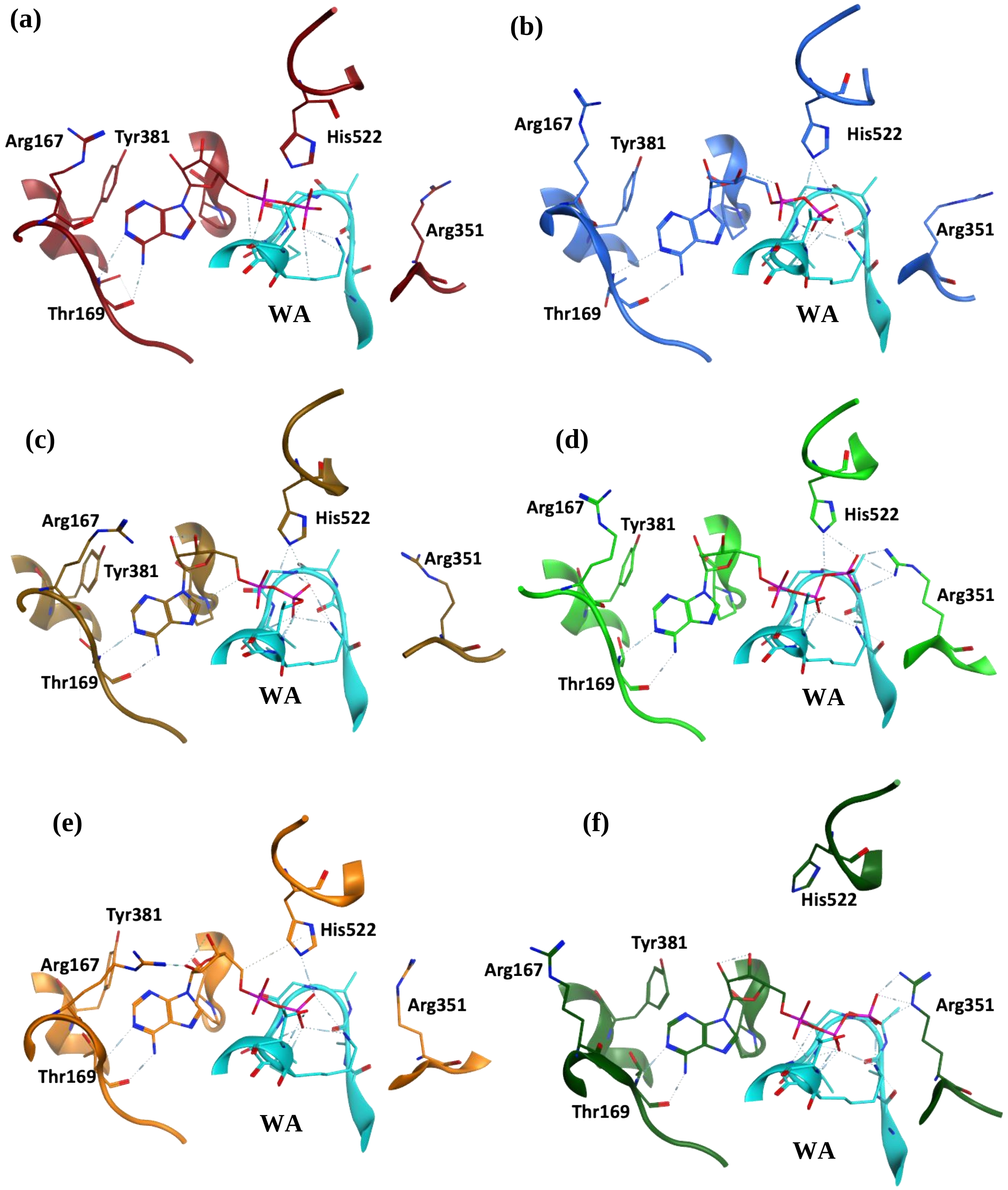

Figure S7 Time series of backbone RMSD for PYD (red), NACHT (purple) and LRR (cyan) over combined 1600 ns MD/AMD simulation of (a) ADP-bound NLRP3 and (b) ATP-bound NLRP3.

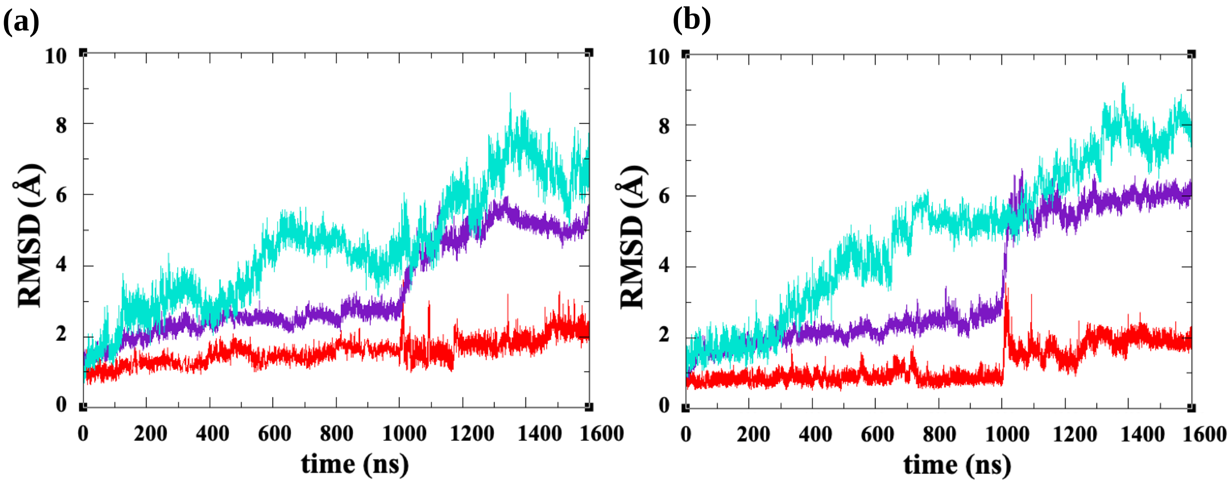

Figure S8 Time series of interatomic distances between (a) Gln308 $O\varepsilon_1$ (from NBD) and Gln480 $N\varepsilon_2$ (from WHD), denoted d_1; (b) Asp305 O (from NBD) and Thr559 $O\gamma_1$ (from HD2) distance, denoted d_2; and (c) Asp312 $O\delta_2$ (from NBD) and Arg556 $N\eta 1$ (from HD2) distance, denoted d_3, for simulation of NLRP3/NEK7 (blue), ADP-bound NLRP3 (maroon) and ATP-bound NLRP3 (dark green). In all cases, the red line represents the distance value in the cryo-EM for d_1 (4.6 Å) and in the initial model for d_2 (7.14 Å) and d_3 (13.7 Å). Note, the residue Thr559 and the side chain of Arg556 are unresolved in the 6NPY cryo-EM structure.

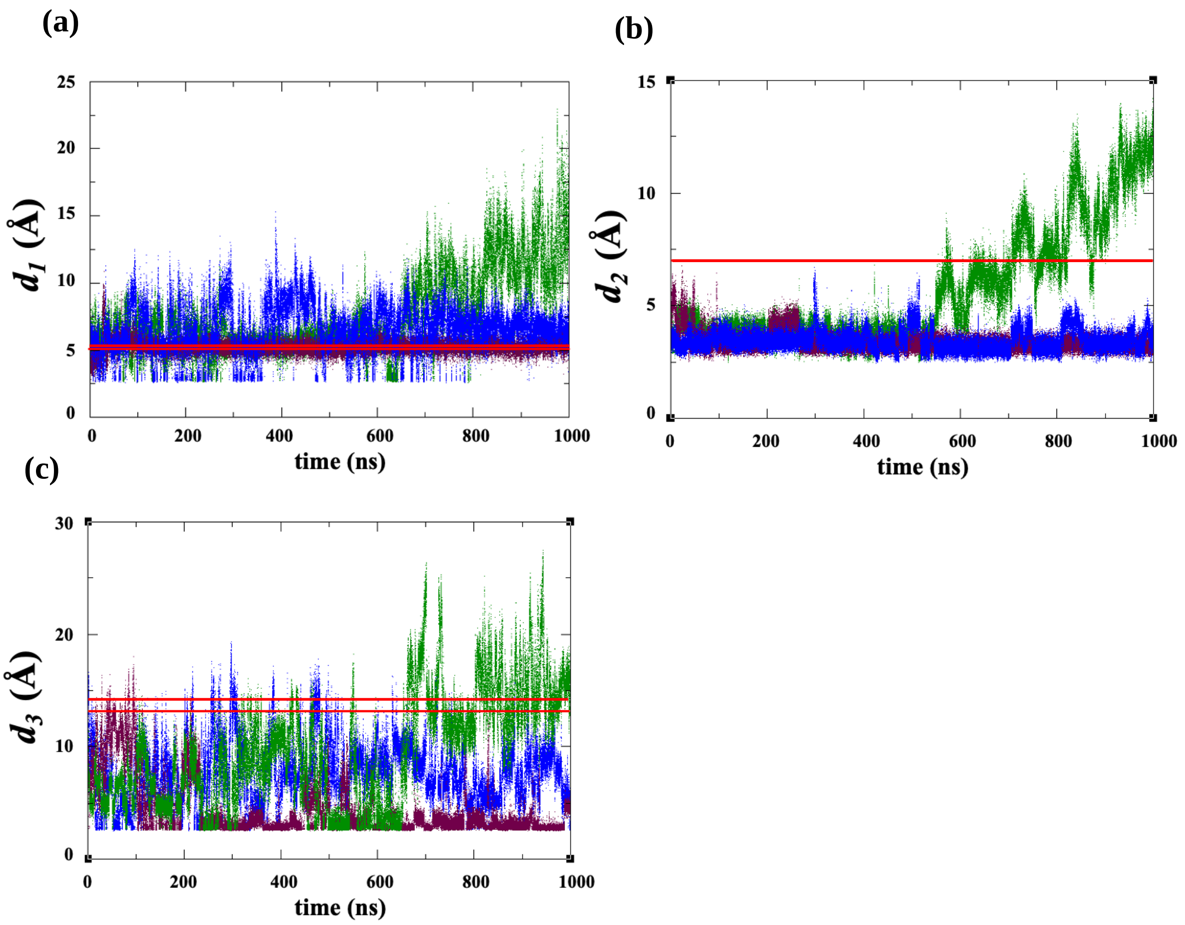

Figure S9 Cofactor accessibility "side-view" between α1 (green) and α2 (cyan) helices of NACHT domain.

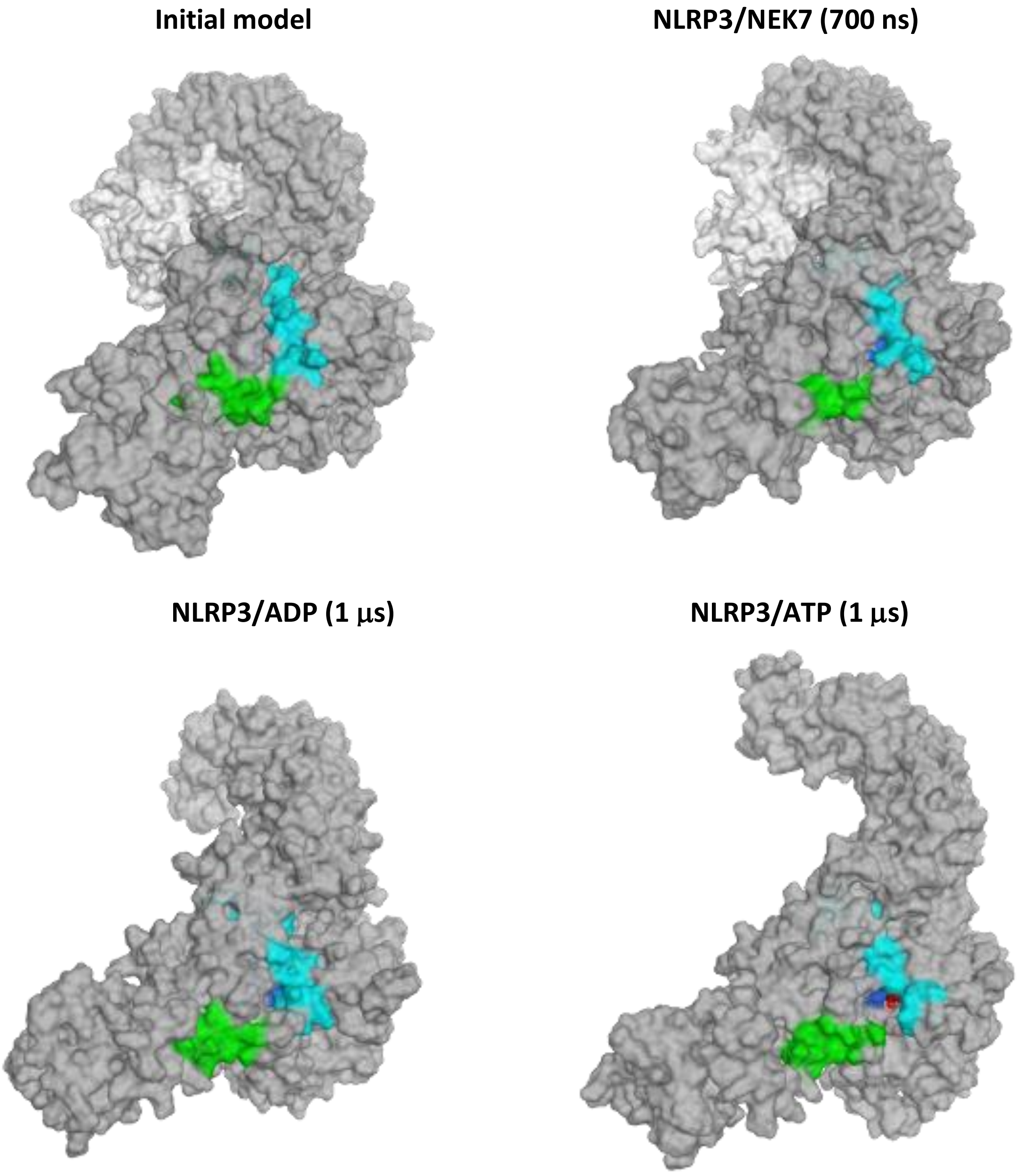

Figure S10 (a) Cofactor accessibility between α1 (green) - α2 (cyan) helices of NACHT domain for ATP/NLRP3 simulation; and between α2 (cyan) - α10 (purple) helices of NACHT domain for ADP/NLRP3 simulation. In all cases, the cofactor inaccessibility is indicated with a cross. Time series of (b) distance d_1 Phe148 – Leu164 Cα-Cα, (c) distance d_2 Val162 – Trp416 Cα-Cα and (d) distance d_3 His260 Cα – Gln526 Cα distances for the microsecond simulation of NLRP3/NEK7 (blue), ADP-bound NLRP3 (maroon) and ATP-bound NLRP3 (dark green). In all cases, the red line represents the distance value in the cryo-EM for d_1 (8.7 Å), d_3 (16.8 Å), and in the initial model for d_2 (7.2 Å). Note, the residue Val162 is unresolved in the cryo-EM.

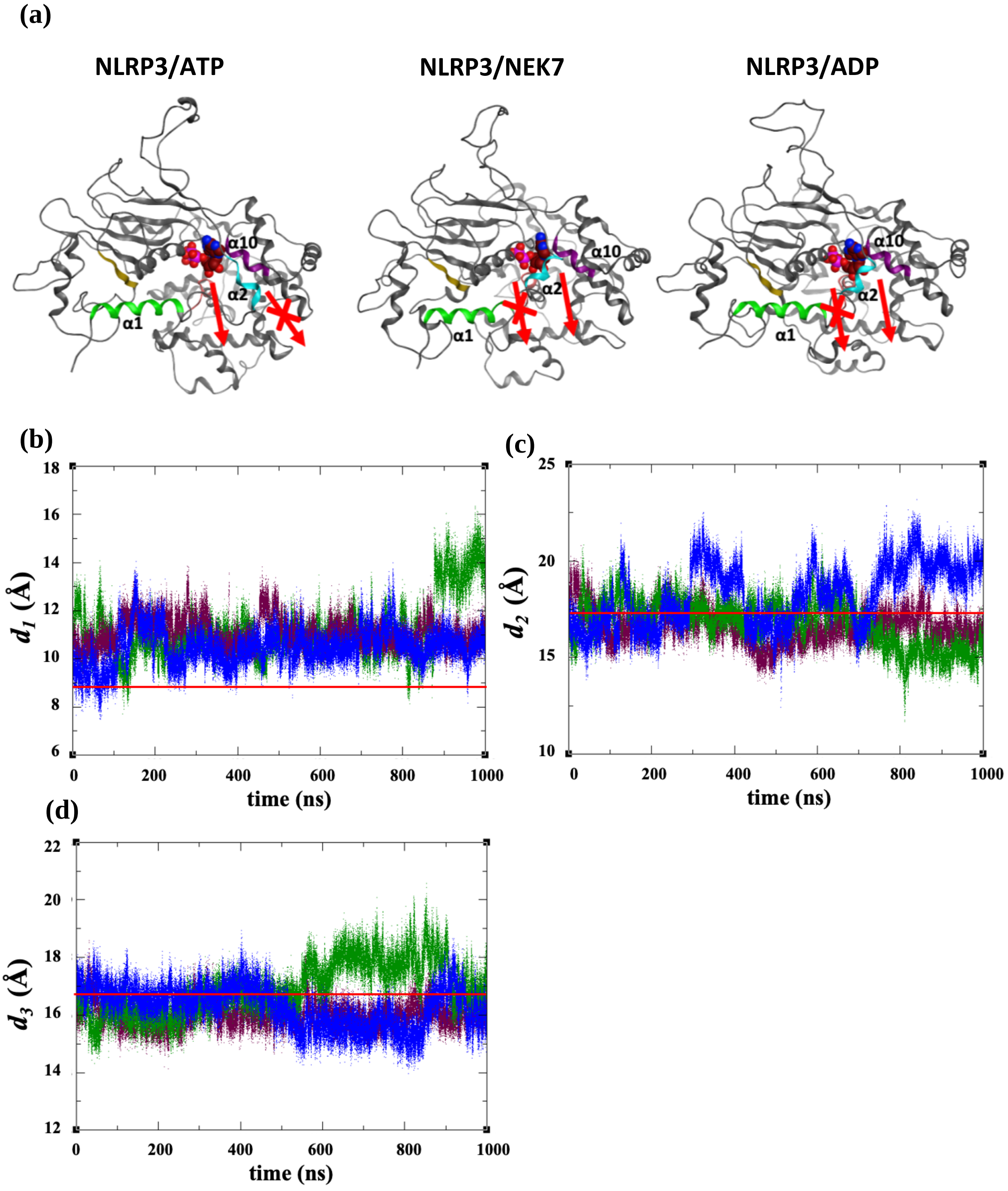

Figure S11 Cofactor accessibility in NLRP3 cage structure. Surface and ribbon representation of the modelled NLRP3 cage structure using the structure at 840 ns of NEK7-bound NLRP3 simulation after deleting NEK7, PYD and PYD-NACHT linker; top view (a,b) and side view (c) showing the cofactor accessibility through channel 2. Cryo-EM structure of inactive NLRP3 oligomer (PDB code 7PZC); top view (d) and side view (e).

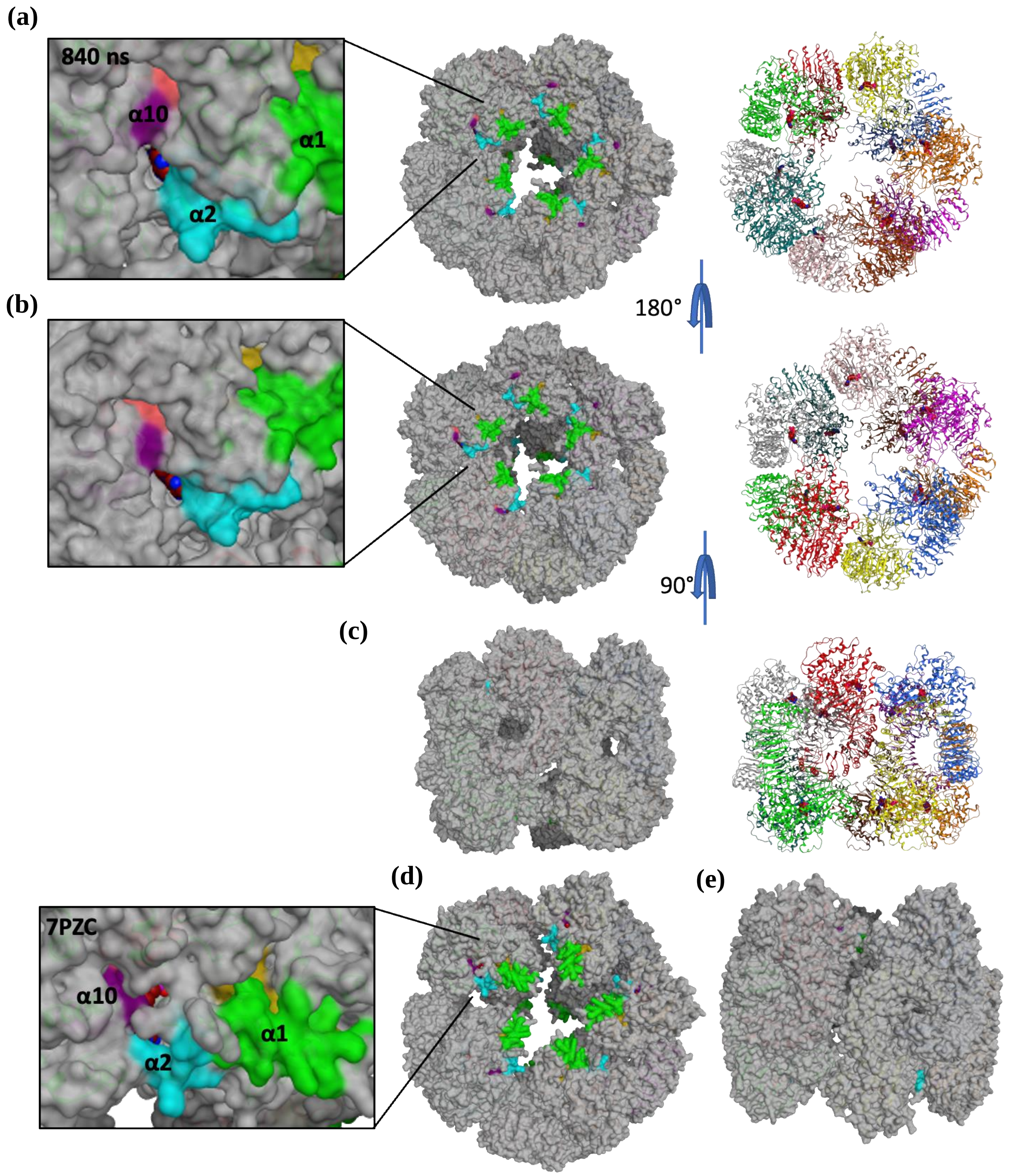

Table S1 AMD parameters for boosting of total potential energy (α_{tot}, E_{tot}) and dihedral energy terms (α_{dih}, E_{dih}). Values in kcal/mol.

parameter	NLRP3/ADP model	NLRP3/ATP model
α_{tot}	138776.1	139036.8
E_{tot}	-2712270.2	-2717091.0
α_{dih}	829.0	829.0
E_{dih}	17371.0	17346.0

Table S2 Average values of backbone RMSD of NLRP3/NEK7 model (in Å) over microsecond MD simulation, with respect to using the initial input structure (labelled "initial") or using the equilibrated structure (labelled "equil"). Standard deviations also shown.

**NLRP3 domains and subdomains**	**sequence (excluding unresolved loops)**	**average RMSD (Å)**			
		**initial**		**equil**	
NACHT-LRR	136 - 1036	4.80	± 0.33	2.55	±0.46
NEK	113 - 300	1.51	± 0.08	0.99	±0.12
PYD	3 - 93	1.42	± 0.32	1.25	±0.37
NACHT	136 - 651	4.36	± 0.16	2.21	±0.33
LRR	700 - 1036	3.17	± 0.21	1.82	±0.27

Table S3 Selected average distances (in Å) between NEK7 and LRR residues for 1000 ns MD trajectory and 6NPY cryo-EM structure. Standard deviations also shown.

**NEK7**		**NLRP3^{LRR}**		**cryo-EM distance (Å)**	**average MD distance (Å)**	
Arg121	$N\eta_2$	Asp807	$O\delta_2$	Sidechain of 807	3.26	±0.58
Arg121	$N\eta_1$	Asp807	$O\delta_1$	unresolved in 6NPY	3.52	±0.78
Arg121	$N\eta_2$	Ser836	$O\gamma$	6.67	3.86	±0.61
Arg121	$N\varepsilon$	Glu864	$O\varepsilon_1$	5.52	4.29	±1.32
Gln129	$N\varepsilon_2$	Asp804	$O\delta_2$	5.58	3.91	±0.75
Arg131	$N\eta_2$	Trp776	$N\varepsilon_1$	5.56	4.06	±0.48
Arg131	$N\eta_1$	Asp747	$O\delta_1$	5.42	3.68	±0.99
Arg136	$N\eta_1$	His724	$N\delta_1$	5.33	3.26	±0.73
Met122	$S\delta$	Arg779	$N\eta_2$	Sidechain of 779	5.90	±1.75
Tyr141	$O\eta$	Arg779	$N\ \eta_1$	unresolved in 6NPY	3.95	±0.97
Tyr141	$O\eta$	Asp750	$O\delta_1$	6.03	5.23	±1.02
Lys140	$N\varsigma$	Asp750	O	5.85	4.72	±0.76
Gln129	$C\gamma$	Trp833	$C\varsigma_2$	3.51	3.93	±0.65
Arg131	$C\delta$	Trp776	$C\varsigma_2$	4.45	4.38	±0.54
Arg136	$C\varsigma$	Leu725	$C\gamma$	9.62	4.89	±0.56
Arg121	$C\alpha$	Glu864	$C\alpha$	11.04	10.68	±1.27
Gln129	$C\alpha$	Asp804	$C\alpha$	10.25	9.72	±0.39
Gln129	$C\alpha$	Trp833	$C\alpha$	9.62	10.18	±0.54
Gln129	$C\alpha$	Arg774	$C\alpha$	12.14	11.50	±0.52
Arg131	$C\alpha$	Asp747	$C\alpha$	11.39	11.49	±0.63
Arg131	$C\alpha$	Trp776	$C\alpha$	10.56	11.57	±0.56
Arg136	$C\alpha$	Leu720	$C\alpha$	17.01	15.70	±0.69
Arg136	$C\alpha$	Asp747	$C\alpha$	12.75	13.85	±0.35
Tyr141	$C\alpha$	Asp750	$C\alpha$	11.51	11.80	±0.56
Met122	$C\alpha$	Arg779	$C\alpha$	12.27	11.33	±0.44
Phe126	$C\alpha$	Trp776	$C\alpha$	11.79	11.26	±0.40
Ile133	$C\alpha$	Asp750	$C\alpha$	10.93	11.51	±0.53

Table S4 Selected average distances (in Å) between NEK7 and HD2 residues for 1000 ns MD trajectory and 6NPY cryo-EM structure. Standard deviations also shown.

**NEK7**		**NLRP3^{HD2}**		**cryo-EM distance (Å)**	**average MD distance (Å)**	
Ser260	Oγ	Glu636	$O\varepsilon_2$	4.81	6.21	±1.69
Ser260	Oγ	Glu636	O	3.59	3.50	±0.96
Ser260	Oγ	Met637	Sδ	8.62	5.85	±1.79
Ser260	O	Met637	N	3.55	4.56	±0.32
Ser260	O	Gln638	N	4.46	2.99	±0.21
Asp261	$O\delta_2$	Glu636	N	5.22	6.04	±1.16
Asp261	$O\delta_1$	Met637	N	4.44	6.17	±0.92
Glu265	N	Gln638	O	2.59	4.07	±0.72
Arg268	$N\eta_2$	Gln638	O	4.30	3.06	±0.42
Glu265	Cβ	Glu639	Cβ	4.72	4.37	±0.51
Glu266	Cβ	Asp641	Cβ	5.97	5.68	±0.73
Ser260	Cα	Gln638	Cα	6.59	6.21	±0.22
Ser260	Cα	Glu636	Cα	5.39	6.60	±0.51
Ser260	Cα	Met637	Cα	5.86	4.93	±0.26
Asp261	Cα	Met637	Cα	6.44	4.78	±0.45
Asp261	Cα	Glu636	Cα	6.08	5.87	±0.62
Glu266	Cα	Asp641	Cα	8.04	6.94	±0.64
Glu265	Cα	Gln638	Cα	5.67	7.07	±0.59

Table S5 Selected average distances (in Å) between NEK7 and NBD residues for 1000 ns MD trajectory and 6NPY cryo-EM structure. Standard deviations also shown.

NEK7		$NLRP3^{NBD}$		cryo-EM distance (Å)	average MD distance (Å)	
Asp290	$O\delta_2$	His364	N	5.25	5.58	±1.22
Lys293	$N\varsigma$	Asp363	$O\delta_2$	10.88	4.99	±2.12
Arg294	$N\eta_1$	Glu356	$O\varepsilon_2$	10.45	7.76	±2.90
Arg294	$N\eta_1$	Glu356	$O\varepsilon_1$	9.60	7.70	±2.87
Arg294	$N\eta_2$	Gln359	$O\varepsilon_1$	4.52	5.43	±1.88
Asp290	$C\beta$	His364	$C\beta$	6.88	6.70	±0.91
Arg294	$C\delta$	Leu355	$C\delta_2$	3.77	5.98	±1.72
Arg294	$C\delta$	Gln359	$C\beta$	6.05	4.69	±1.14
Arg294	$C\gamma$	Leu355	$C\beta$	6.28	5.94	±1.41
Asp290	$C\alpha$	His364	$C\alpha$	7.41	6.95	±0.76
Asp290	$C\alpha$	Gln359	$C\alpha$	8.51	9.42	±1.20
Lys293	$C\alpha$	Pro365	$C\alpha$	10.74	9.56	±0.46

Table S6 Selected average interatomic distances (in Å) between cofactor (ADP / ATP) and NLRP3 binding site residues from simulation of NLRP3/NEK7, ADP-bound and ATP-bound NLRP3 over 1000 ns MD simulation and for 6NPY cryo-EM structure. Standard deviations also shown.

**ligand atom name**	**protein residue number**		**distance in cryo-EM (Å)**	**average MD distance (Å)**					
				**NLRP3/NEK7 /ADP model**		**NLRP3/ADP model**		**NLRP3/ATP model**	
O1β	Lys232	Nς	3.60	3.42	±0.22	3.65	±0.38	2.77	±0.11
O1β	Thr233	N	2.44	3.06	±0.19	3.63	±0.96	4.38	±0.21
O1β	His522	$N\varepsilon_2$	4.63	4.54	±0.20	4.00	±0.67	7.34	±3.16
O2β	His522	$N\varepsilon_2$	3.12	3.21	±0.31	3.54	±0.84	6.99	±2.91
O3β	Gly229	N	4.01	3.65	±0.23	4.08	±0.61	3.35	±0.26
O3β	His522	$N\varepsilon_2$	3.32	4.80	±0.19	4.80	±0.37	5.16	±3.26
O3α	Gly229	N	4.95	3.96	±0.23	3.78	±0.26	4.24	±0.24
O1α	Gly231	N	4.10	3.73	±0.21	3.80	±0.29	5.64	±0.21
O1α	Lys232	N	3.20	3.22	±0.17	3.42	±0.22	5.42	±0.18
O1α	Ile234	N	2.46	2.87	±0.12	2.95	±0.14	4.58	±0.32
O5`	Ile230	N	5.31	3.92	±0.28	4.90	±0.38	5.27	±0.35
O5`	Gly231	N	3.84	3.25	±0.20	4.21	±0.30	4.31	±0.28
O2`	Trp416	N	4.95	6.71	±0.78	6.36	±0.95	5.91	±0.47
O3`	Leu413	N	5.77	6.74	±0.36	6.48	±0.44	6.33	±0.39
O4`	Tyr381	Oη	6.86	5.76	±0.93	6.77	±0.67	6.72	±0.46
C2	Phe373	Cς	4.79	4.99	±0.76	4.32	±0.48	4.28	±0.45
C2	Tyr381	Cβ	6.01	6.43	±0.59	6.51	±0.59	6.54	±0.52
C2	Tyr381	$C\delta_1$	3.85	4.35	±0.47	4.98	±0.97	4.37	±0.43
C4	Pro412	Cβ	4.46	4.54	±0.34	3.82	±0.27	3.86	±0.26
C8	Pro412	Cγ	4.03	4.05	±0.36	4.11	±0.36	4.18	±0.32
N1	Arg167	O	3.00	4.17	±0.31	3.85	±0.43	4.03	±0.39
N1	Thr169	$O\gamma_1$	4.04	5.67	±0.53	4.38	±0.94	4.72	±0.88
N3	Tyr381	Oη	4.68	4.68	±0.57	4.93	±0.42	4.86	±0.28
N6	Thr169	O	2.92	2.90	±0.16	2.94	±0.16	2.92	±0.15

Table S7 Average values of RMSD (in Å) of ADP-bound and ATP-bound NLRP3 from 1000 ns MD and further 600 ns AMD. RMSD was calculated using as reference either the initial input structure (i.e NLRP3/NEK7 at 700 ns, labelled "initial") or using the equilibrated structure (i.e. after 50 ns of equilibration of the NEK7-free system, labelled "equil"). Standard deviations also shown.

		**reference**	**average RMSD (Å)**			
			**NACHT-LRR**	**PYD**	**NACHT**	**LRR**
**NLRP3/ADP**	**MD**	**initial**	4.58 ±1.02	1.56 ±0.21	2.41 ±0.32	3.35 ±0.95
		**equil**	4.16 ±0.87	1.39 ±0.24	2.34 ±0.39	3.52 ±1.06
	**AMD**	**initial**	9.15 ±2.13	1.80 ±0.27	4.93 ±0.47	5.81 ±0.96
		**equil**	8.36 ±1.55	1.84 ±0.32	4.92 ±0.55	6.12 ±1.02
**NLRP3/ATP**	**MD**	**initial**	5.24 ±1.63	1.33 ±0.16	2.08 ±0.39	3.54 ±1.51
		**equil**	5.31 ±1.62	0.86 ±0.14	2.13 ±0.38	3.63 ±1.49
	**AMD**	**initial**	9.90 ±1.22	1.86 ±0.28	5.82 ±0.51	6.84 ±0.90
		**equil**	10.14 ±1.28	1.78 ±0.29	5.73 ±0.45	7.02 ±0.94

Video descriptions:

Visualization of MD trajectories over microsecond MD simulation of ADP-bound NLRP3/NEK7 (**Video S1**) and over combined 1600 ns MD/AMD simulation of ADP-bound NLRP3 (**Video S2**) and ATP-bound NLRP3 (**Video S3**). Note, NBD-HD1 subdomains are superimposed over all frames.

Principal component analysis of trajectories: Animations of motion along principal eigenvectors from microsecond MD of NLRP3/NEK7 (**Video S4**), and from combined 1600 ns MD/AMD simulation of ADP-bound NLRP3 (**Video S5**) and ATP-bound NLRP3 (**Video S6**). Note that the subdomains are colored using the same scheme as in Figure 1c.

Video S7 Principal component analysis of trajectories: Animations of movement along principal eigenvectors from aggregate of microsecond MD of NLRP3/NEK7; and 1600 ns MD/AMD simulation of ADP-bound NLRP3 and ATP-bound NLRP3. Note that the subdomains are colored using the same scheme as in Figure 1c.
